# A synthesis of soil carbon and nitrogen recovery after wetland restoration and creation in the United States

**DOI:** 10.1038/s41598-017-08511-y

**Published:** 2017-08-11

**Authors:** Lingfei Yu, Yao Huang, Feifei Sun, Wenjuan Sun

**Affiliations:** State Key Laboratory of Vegetation and Environmental Change, Institute of Botany, Chinese Academy of Sciences, Beijing, 100093 PR China

## Abstract

Wetland restoration and creation efforts have been widely attempted as a way to compensate for wetland losses and to recover wetland functions; however, to date, there has been no comprehensive evaluation of the efficacy of soil carbon (C) and nitrogen (N) content recovery at a regional scale. This meta-analysis synthesizes 48 articles to identify the general patterns of soil C and N change after wetland restoration and creation in the United States. Our results indicate that, after 11–20 years, soil C and N in restored and created wetlands are still significantly lower by 51.7% and 50.3%, respectively, than those in natural wetlands. The soil C and N in restored wetlands recovered faster than in created wetlands. Furthermore, the soil C in restored organic flat and created depressional wetlands recovered more rapidly than in restored and created hydrologically open wetlands (riverine and tidal), respectively. Mean annual temperature and soil texture were recognized as two crucial abiotic factors affecting soil C and N recovery. Linear regression analysis revealed a positive relationship between the restoration and creation effect sizes on soil C and N, indicating that wetlands may alleviate N limitations intrinsically during C recovery processes.

## Introduction

Natural wetlands store a large amount of carbon (C), with 202–377 Gt C stored in the top 100 cm of soil^1, 2^; however, due to human activities, over half of the world’s wetlands have been lost during the 20th century^3, 4^. The primary causes of wetland loss include draining and filling for agriculture as well as human settlements. Aerobic conditions in degraded wetlands accelerate microbial respiration, result in the oxidation of accumulated organic C and lead to a net release of C into the atmosphere^5^, which has strongly influenced atmospheric CO_2_ levels and global C balances.

The Ramsar Convention, signed in 1971, is an intergovernmental treaty that provides the framework for national action and international cooperation in the conservation and sustainable use of wetlands and their resources. To date, more than 2 × 10^8^ ha of wetlands at 2178 sites around the world have been designated Wetlands of International Importance. Following the convention, many countries have implemented wetland conservation and restoration projects. In North America alone, over $70 billion (U.S. dollars) have been spent attempting to restore more than 3 × 10^6^ ha of wetlands in the last 20 years^6, 7^.

The restoration of degraded wetlands and the creation of new ones has been undertaken with the objective of recovering their physical, chemical and biological processes and properties^8^. In terms of soil C stocks, when wetland hydrology regimes are restored, plant litter and root biomass and external riverine/tidal inputs of organic particles provide the primary inputs of organic C into soils^9, 10^, and anaerobic wetland soils enable organic C to accumulate slowly; however, it is still unclear whether or when these restored and created wetlands attain the C levels of natural wetland soils. Some studies have found that restored and created wetlands can develop soil C and soil organic matter (SOM) conditions comparable to those in natural wetlands within7–20 years following restoration or creation^11–13^. However, many studies have demonstrated that restoration and creation do not necessarily recover the soil organic C or SOM of the degraded wetlands to pre-disturbance levels within a certain period^14–17^. Some studies of individual sites have indicated that the restored and created wetlands may never achieve soil C levels equivalent to natural wetlands^18, 19^. Assuming there is a trajectory of C accumulation, several studies have estimated that it would take 15–300 years for soil C pools to reach the levels observed in natural wetlands when based on several different models^15, 20–22^. Moreno-Mateos *et al*.^6^ conducted a meta-analysis to examine the temporal change in soil C storage in restored and created wetlands throughout the world, and found that the level had not recovered to natural levels after 20 years.

As one of the limiting nutrients in terrestrial ecosystems, nitrogen (N) controls primary production in the biosphere and plays a crucial role in the C cycle^23^. Aerobic conditions in artificially drained wetlands also perturb N storage and cycling, allowing for the mineralization of organic N^3^. Inorganic N is rapidly processed by microorganisms and plants, leaving the original pool of N in the soil depleted or unavailable. Although wetland restoration and creation have been reported to accrue soil N to some extent, soil N does not necessarily reach the original levels after a set number of years^18, 24, 25^. Some studies at single sites have predicted that it will take 2–40 years for restored and created wetlands to develop soil N levels equivalent to natural wetlands^18, 20, 26^.

Research investigating the drivers and constraints of wetland recovery is critical for improving restoration techniques and the management of natural wetlands^27^. Wetland recovery might be affected by the physical characteristics of wetlands, land-use history, restoration activities, topography, and environmental factors^6, 27–30^. It has been suggested that the initial recovery rate may be dependent on the degree of openness of a wetland and its connectivity to external sources, e.g., sediment, organic matter, seeds, and rhizomes^16^. The hydrologic open systems such as riverine and tidal wetlands, are linked to larger hydrologic regimes through natural flow variation; therefore, external inputs of mineral sediment and organic particles may accelerate soil development^10^. In contrast, in relatively isolated systems such as organic flat and depressional wetlands, the dominant sources of water are precipitation or groundwater discharge, with minimal external source inputs; thus, their soil development is considered comparably slow. It would also be expected that warm temperatures could prompt soil development after wetland restoration and creation because of accelerated ecosystem processes^31^, e.g., increased net primary production (NPP) and plant above- and below-ground C pools in wetland ecosystems. Soil texture has been considered a crucial factor affecting the soil C and N content^32, 33^. Therefore, it is assumed that soil texture also plays an important role in the process of soil C and N recovery. These factors most likely determine the overall efficacy of wetland restoration and creation projects. However, there is little plot-based data on how soil C and N recover across extensive environment conditions (hydrogeomorphic type, climate and soil texture).

Since the 1950s, an extensive area of wetlands was lost due to silviculture, salt water intrusion, agriculture, and urban and rural development in the conterminous United States^34–38^. However, the average annual surface loss rate between 2004 and 2009 declined by 97% from the previous period (1950s–1970s), which has been attributed to wetland restoration and creation^36^. Many individual studies have investigated the trajectories and drivers of soil C and N recovery after wetland restoration or creation, whereas less attention has been paid to an overall evaluation of soil C and N dynamics at a regional scale. Therefore, we compiled data from 48 individual studies to identify the general patterns of soil C and N change after wetland restoration and creation in the United States. These results were obtained from studies of the recovery age, hydrogeomorphic type, soil-sampling depth, soil texture and climate at the study sites. The objective of this study was to address the following questions. First, to what extent do soil C and N approach the levels observed in natural wetlands? Second, how do soil C and N change along the age of restoration and creation? Finally, how do recovery approaches (restoration and creation), hydrogeomorphic type, soil sampling depth and other abiotic factors (climate and soil texture) affect soil C and N recovery?

## Results

### The recovery of soil C, N and other soil properties

**Figure 1:**
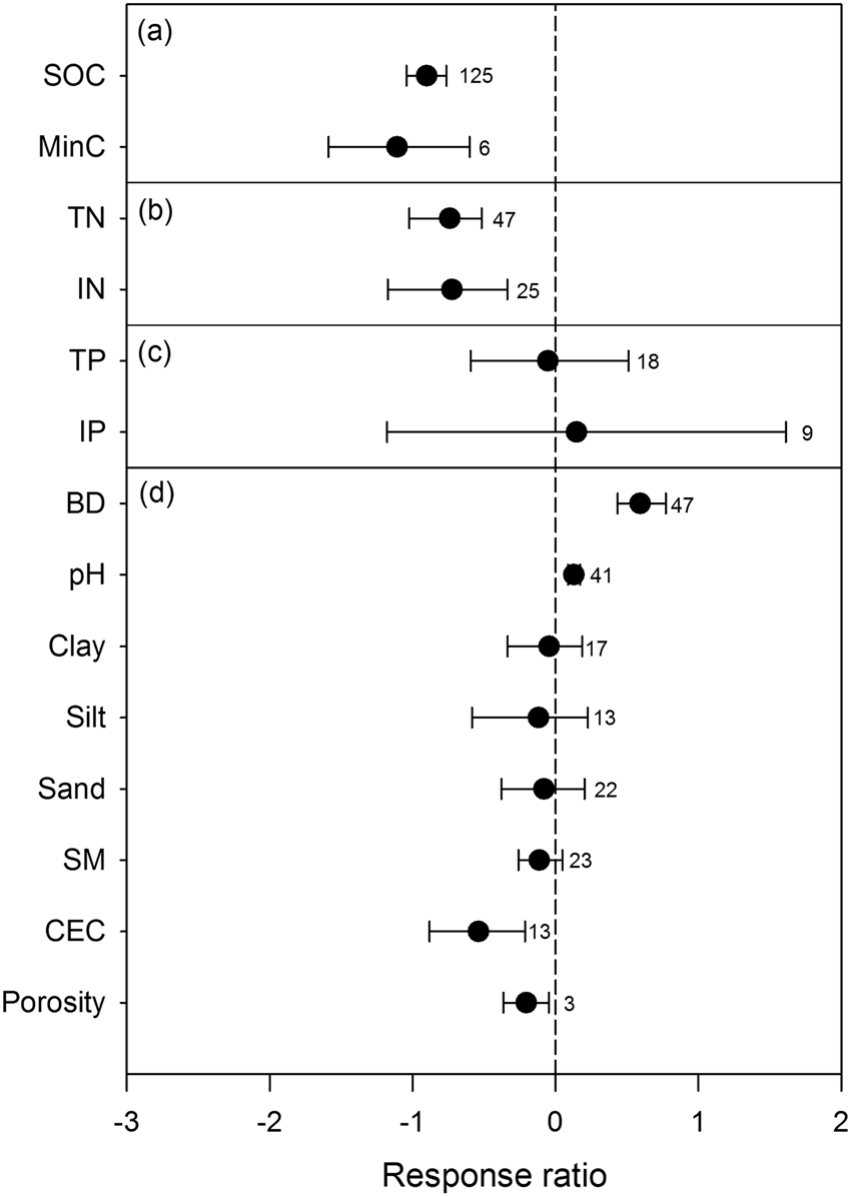
Mean response ratios (RR_++_) of soil variables related to carbon (**a**), nitrogen (**b**), phosphorus (**c**) and other properties (**d**) after wetland restoration and creation. The data represent the means ±95% confidence intervals. The dashed line indicates a mean response ratio of 0. The values of soil variables in the restored and created wetlands were considered significantly higher (RR_++_ > 0) or lower (RR_++_ < 0) than those in the natural wetland if the 95% CI of RR_++_ for the variables did not cover zero. The sample size for each variable is shown next to the relevant point. SOC, soil organic carbon; MinC, mineralizable carbon; TN, total nitrogen; IN, inorganic nitrogen; TP, total phosphorus; IP, inorganic phosphorus; BD, bulk density; SM, soil moisture; and CEC, cation exchange capacity.

The changes observed in 14 variables related to soil properties indicated different responses to wetland restoration and creation (Fig. 1). Compared to natural wetlands, soil organic carbon (SOC) and mineralizable C were significantly lower, by 59.4% and 67.0% in restored and created wetlands, respectively (Fig. 1a, Table S1). The total soil N and inorganic N were significantly lower, by 52.3% and 51.5%, respectively (Fig. 1b, Table S1). The total phosphorus (P) and inorganic P decreased and increased only slightly in restored and created wetlands, respectively, but they exhibited no significant differences from natural wetlands (Fig. 1c, Table S1). Compared to natural wetlands, the soil bulk density and pH significantly increased, by 81.0% and 14.0%, respectively, and the soil texture (clay, silt and sand) and soil moisture exhibited an insignificant difference; the cation exchange capacity (CEC) and porosity were still significantly lower, by 41.7% and 18.5%, respectively, in restored and created wetlands (Fig. 1d, Table S1).

### Factors affecting soil C and N recovery

**Figure 2:**
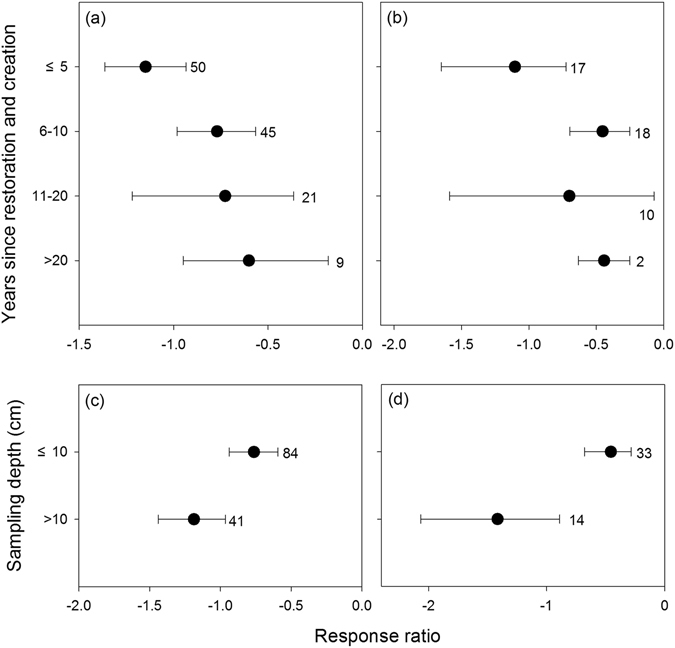
Soil C (**a**,**c**) and N (**b**,**d**) recovery at different ages after restoration and creation (**a**,**b**) and at different soil sampling depths (**c**,**d**). The SOC or total N in restored and created wetland was considered significantly higher (RR_++_ > 0) or lower (RR_++_ < 0) than that in the natural wetland if its 95% CI of RR_++_ did not cover zero. The sample size for each variable is shown next to the relevant point.

Among the four wetland age categories, soil C and N generally increased with increasing recovery age; however, soil C and N in restored and created wetlands were still significantly lower, by 51.7% and 50.3%, than in natural wetlands after 11–20 years (Fig. 2a,b). The recovery of soil C and N differed significantly between ≤10 cm and >10 cm soil depths (between-class heterogeneity (Q_B_) = 4.96, *p* < 0.01 for soil C; Q_B_ = 9.07, *p* < 0.001 for soil N; Fig. 2c,d). The average values of soil C and N in restored and created wetlands were 46.6% and 63.5% of the values in natural wetlands at ≤10 cm, 30.5% and 24.3% at >10 cm soil depths, respectively.

**Figure 3:**
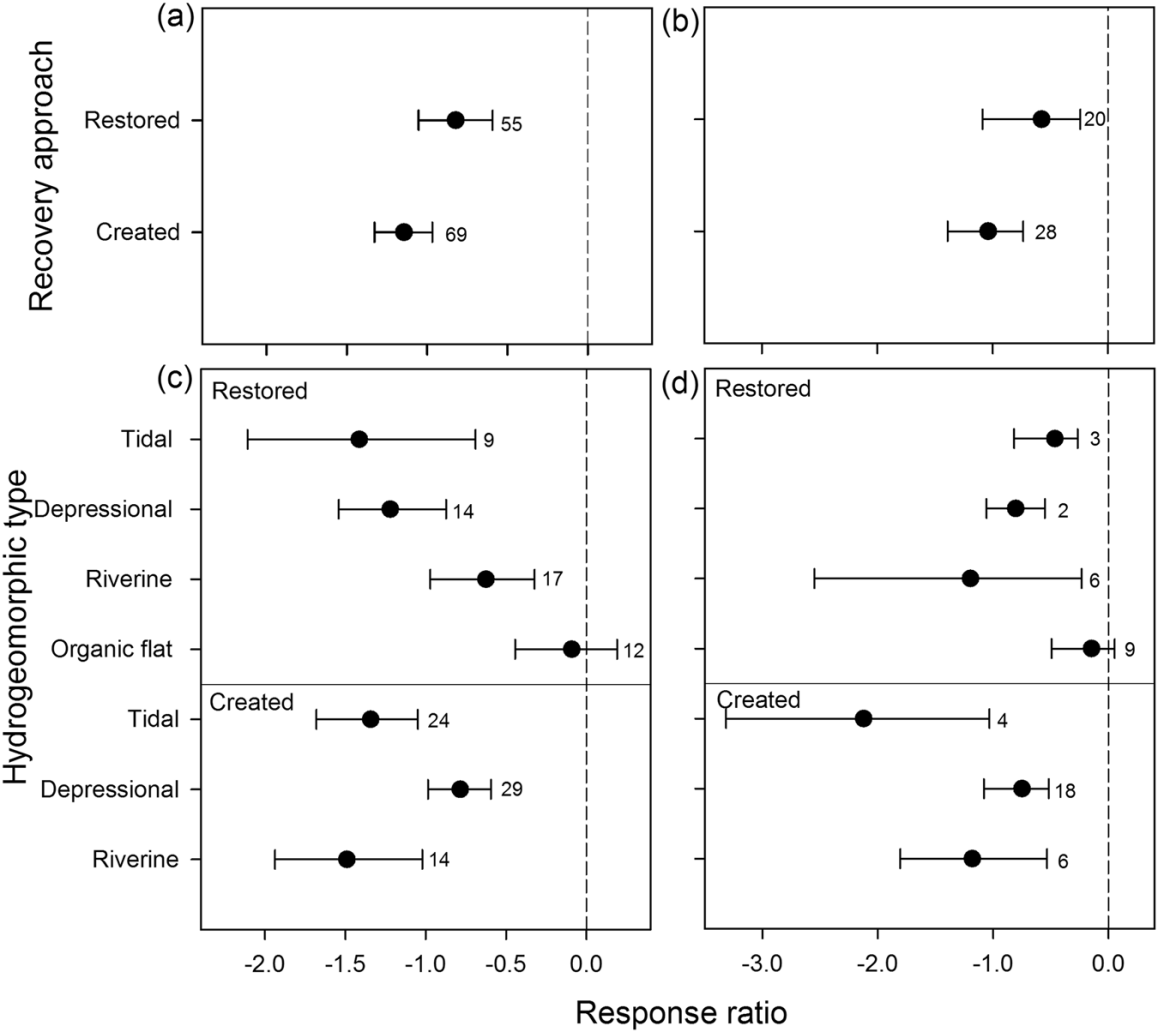
The effects of recovery approach and hydrogeomorphic type on SOC (**a**,**c**) and total N (**b**,**d**) recovery after wetland restoration and creation. The Data represent the means ± 95% confidence intervals. The dashed line indicates a mean response ratio (RR_++_) of 0. The SOC or total N in restored and created wetlands was considered significantly higher (RR_++_ > 0) or lower (RR_++_ < 0) than that in the natural wetland if its 95% CI of RR_++_ did not cover zero. The sample size for each variable is shown next to the relevant point.

We investigated the soil C and N recovery under different recovery approaches and hydrogeomorphic types (Fig. 3). The mean response ratio (RR_++_) of soil C and N in restored wetland was significantly higher than that in created wetland (Q_B_ = 3.16, *p* < 0.05 for soil C; Q_B_ = 2.49, *p* < 0.1 for soil N; Fig. 3a,b), indicating that wetland restoration was more effective for soil C and N recovery than wetland creation. The RR_++_ of soil C varied significantly among hydrogeomorphic types in both restored and created wetlands (Q_B_ = 12.41, *p* < 0.001 for restored wetland; Q_B_ = 6.35, *p* < 0.01 for created wetland; Fig. 3c). Restored organic flat wetlands had the largest RR_++_ of soil C, followed by riverine, depressional and tidal wetlands, whereas the RR_++_ of soil C among created wetlands was highest in depressional wetlands, followed by tidal and riverine wetlands. The sample size was small in the study of soil N recovery among different hydrogeomorphic types after wetland restoration or creation. Based on the limited data, we found a significant difference in soil N recovery among the hydrogeomorphic types in created wetlands (Q_B_ = 6.33, *p* < 0.05), but no significant difference existed in restored wetlands (Fig. 3d).

**Figure 4:**
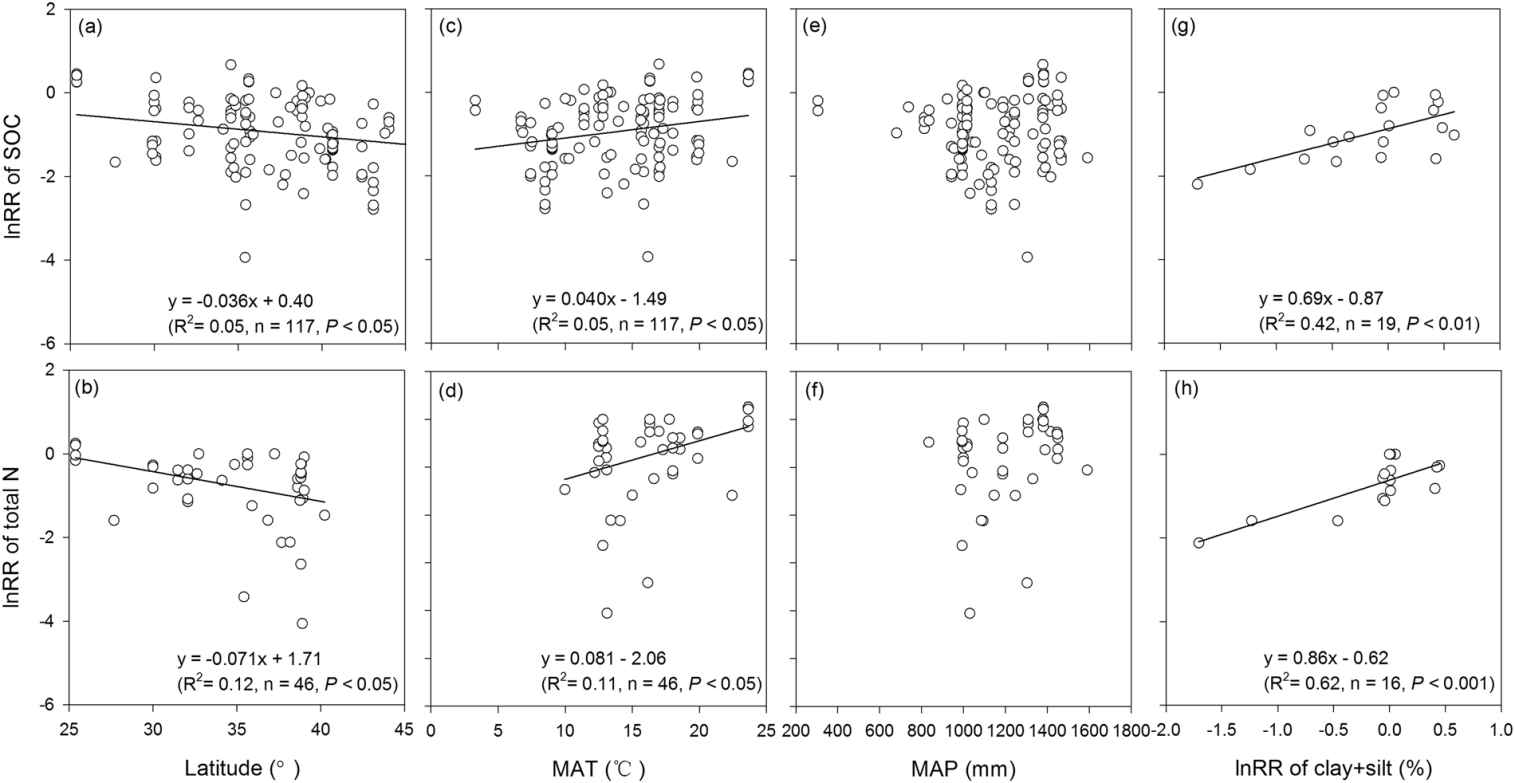
Relationships of the response ratios (lnRR) of SOC (**a**,**c**,**e**,**g**) and total N (**b**,**d**,**f**,**h**) with latitude (**a**,**b**), mean annual temperature (**c**,**d**), mean annual precipitation (**e**,**f**) and lnRR of clay + silt (**g**,**h**). The lnRR > 0 indicates that the SOC or total N in restored and created wetland was higher than that in the natural wetland, whereas lnRR < 0 indicates that the SOC or total N was relatively lower than that in the natural wetland.

Geographically, the response ratios (lnRR) of soil C and N exhibited a significant decreasing trend with latitude (Fig. 4a,b). Among the climate factors, the lnRR of soil C and N significantly increased with mean annual temperature (Fig. 4c,d) but had no relationship with the mean annual precipitation (Fig. 4e,f). Furthermore, the lnRR of the soil C and N were also positively correlated with the clay + silt content (Fig. 4g,h). It is notable that the lnRR of soil N had a steeper slope against latitude, mean annual temperature and clay + silt than the lnRR of soil C, suggesting that soil N recovery may be more sensitive to environmental change than soil C.

### Relationship of soil C to N recovery

**Figure 5:**
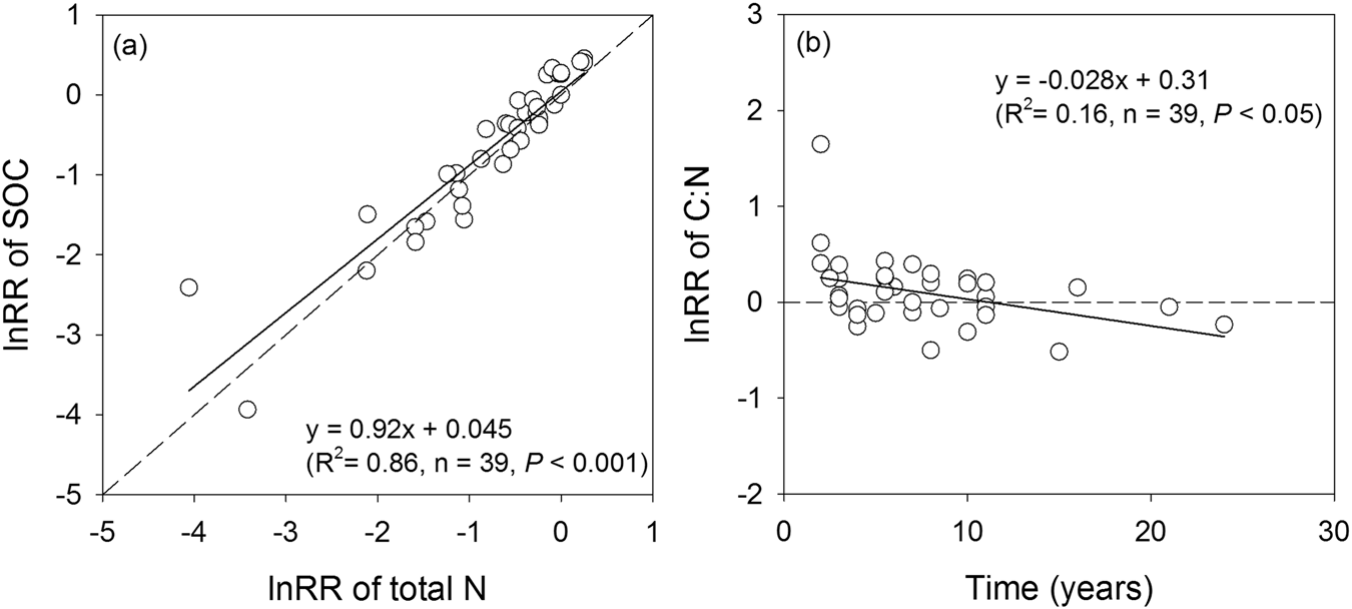
Relationships between the response ratios (lnRR) of SOC and total N (**a**), and between the response ratios of soil C:N and years since restoration and creation (**b**). The dashed line in panels (**a**) and (**b**) represent the 1:1 line and a mean response ratio of 0, respectively. An lnRR > 0 indicates that the SOC or total N in restored and created wetland was higher than that in the natural wetland, whereas lnRR < 0 indicates that the SOC or total N was relatively lower than that in the natural wetland.

Linear-regression analysis revealed a positive relationship between the lnRR of soil C and N (Fig. 5a), suggesting that the recovery of soil C is accompanied by soil N accumulation. The slope (0.92) was significantly different from 1 (*P* < 0.05), implying that the relative change in soil N was larger than that of soil C. Consequently, the lnRR of the soil C:N ratio illustrated a significantly negative relationship with recovery age (Fig. 5b).

## Discussion

The aerobic conditions of degraded wetlands accelerated the mineralization of soil organic matter, leading to the original pool of C and N in the soil being depleted^3, 5^. After wetlands are restored or created, more anaerobic conditions allow for stores of organic matter to re-accumulate slowly in the soil. Although soil C and N increased over time, their average values were still only 48.4% and 49.7% of the value in natural wetlands 11–20 years following restoration and creation (Fig. 2a,b). This result was comparable to the findings reported by Moreno-Mateos *et al*.^6^, who used meta-analysis to survey the biogeochemical recovery of restored and created wetlands worldwide and found that C and N storage did not reach a natural level within 20 years following restoration. Unfortunately, most of the data that we compiled described projects ≤15 years old (Fig. S1a,b), and a lack of long-term data prevents us from accessing whether or when soil C and N could converge with those in natural wetlands. It has been reported that mitigation projects involving freshwater marshes should allow at least 15–20 years before their success can be judged, and restoration and creation of forested wetlands, coastal wetlands, or peatlands may require even more time^39^. Thus, data concerning the long-term recovery of wetlands are needed to confirm the success of these efforts. In contrast to soil C and N, soil P in restored and created wetlands exhibited no significant difference from that in natural wetlands (Fig. 1c). This lack of difference may be due to the conservative cycling of soil P. As reported by Smil^40^, P lacks an exchange with the atmosphere because it does not form any long-living gaseous compounds.

The magnitude of soil C and N recovery at ≤10 cm depths were larger than at >10 cm soil depths (Fig. 2c,d). This result may be related to the difference in soil C or N accumulation across soil depths. The soil C storage occurs as a result of plant production and external inputs of organic particles introduced through the hydrology of wetland systems^10^. The majority of plant production (e.g., roots, buried stems, and litterfall), except the roots in forested wetlands, and mineral and organic matter inputs occur at the soil surface. Soil N storage could be largely derived from atmospheric deposition, biological N fixation and external inputs through hydrology. Furthermore, several created wetlands were amended with organic-rich soil at the surface, potentially accelerating C and N accumulation in the topsoil^41, 42^. Therefore, the rate of C and N accumulation decreased as the soil depth increased and exhibited no significant change in the deep soil after restoration^12, 43^. This implied that more time is needed for the full recovery of C and N in the deep soil layers.

Wetland restoration and creation are increasingly used as strategies to mitigate the loss of wetland function. In this study, it seemed that wetland restoration was more efficient in soil C and N recovery than creation (Fig. 3a,b). The soil properties in restored wetlands may still retain some of the same properties as their natural counterparts (e.g., relatively high soil C and N). In contrast, created wetlands often have relatively low soil C and N as a consequence of construction on organic-poor upland soils. Furthermore, many created wetlands in our synthesis were excavated, and organic-rich topsoil was removed, leaving the subsoil with low C and N.

The hydrogeomorphic type also affected the soil C recovery. Riverine and tidal wetlands, the two hydrologically open systems, were expected to recover soil C faster than isolated organic flats and depressional wetlands due to their external inputs of mineral sediment and organic particles^10, 16^. However, in this study, restored organic flats and created depressional wetlands seemed to recover more rapidly than riverine and tidal wetlands (Fig. 3c). Plant biomass is likely the primary driver of soil development within the recovering isolated systems. The fast recovery of soil C was likely explained by the rapid increase of plant biomass, as documented by Ballantine and Schneider^16^.

Soil C and N accumulation are related to climate through biotic processes associated with vegetation productivity and the decomposition of organic matter^2, 44^. Tropical-subtropical wetlands exhibit, on average, 10 times higher net primary production than high-latitude wetlands^45^; however, warm temperatures can accelerate soil respiration^31^. Therefore, in warm climates, large litter inputs may be offset by rapid decomposition, which may reduce soil C and N accumulation. The present study indicated that the net balance of soil C or N input and output, that is the soil C or N recovery, increased with mean annual temperature (Fig. 4c,d). This finding implied that with an increase in mean annual temperature, the stimulation of plant productivity was greater than litter decomposition, which dominated the C balance of wetlands in the United States. This result is consistent with that of Moreno-Mateos *et al*.^6^, who found that the biogeochemical functions of wetlands that were restored in a tropical climate recovered relatively rapidly compared with those in temperate and cold climates. Mean annual precipitation, another climate factor, was not significantly related to soil C and N recovery (Fig. 4e,f), which was probably due to the water-saturated soil in wetland systems thus causing precipitation to have little effect on the C and N dynamics in wetland soils.

As expected, soil texture had a significant effect on soil C and N recovery, which was positively correlated with the clay + silt content (Fig. 4g,h). Increasing the clay and silt content reduces microbial decomposition through its great ability to stabilize or protect SOM that is introduced to the soil, thus leading to the accumulation of SOM^46, 47^. Furthermore, clay and silt concentrations may improve soil nutrients, which, in turn, increase the C inputs into soils via plant productivity^48^.

Several other factors, e.g., vegetation type, initial land use, and restoration activities, are potentially important for wetland soil C and N recovery. However, we were unable to evaluate their influences because it was difficult to categorize these factors on the basis of the information provided in the studies. For example, according to the dominant vegetation, wetlands were grouped into moss-lichen, emergent, scrub-shrub, and forested wetlands^49^. Many wetlands we reviewed had no information about the vegetation type or dominant plants but were planted with tree or shrub seedlings, so they were hard to categorize.

The global importance of wetlands as carbon sinks is widely recognized^50^. Wetland restoration has been proven to be an effective method of C sequestration in order to mitigate the greenhouse effect^51^. However, whether soil C sequestration after wetland restoration can be sustained is dependent on the availability of N, since additional N is required to support terrestrial C accumulation^52^. Our study indicates that the recovery of soil C after wetland restoration and creation was accompanied by N accumulation due to the strong relationships between soil C and N recovery (Fig. 5a). Soil C accumulations have also been documented in association with N accumulations after land use change in grasslands and forests^52, 53^. N accretion may mitigate N limitation resulting from disturbance, accelerate internal N cycling (i.e., N mineralization) and enable soils to provide more available N for plant productivity^54^. Our study also indicated that, relative to the soil C:N ratio in natural wetlands, the C:N ratio decreased gradually over time in restored and created wetlands (Fig. 5b). This result implied that greater relative increases in soil N pools than in soil C pools would ensure a progressively increased N supply in the long term, which would maintain soil C accumulation during recovery processes.

In summary, our analysis indicates that soil C and N content in restored and created wetlands were significantly lower than those in natural wetlands on a regional scale. Although soil C and N increased over recovery ages, the levels were still only 48.4% and 49.7% of the value in natural wetland 11–20 years following restoration and creation. In the future, more effort is needed to explore the long-term recovery of soil C and N to provide a thorough understanding of their changes following wetland restoration and creation.

## Methods

### Data compilation

We searched peer-reviewed journal articles published before April 2016 in the ISI Web of Knowledge. The search terms were “(wetland or marsh or mire or fen or bog or swamp or peatland or floodplain or mangrove) AND (creat* or re-creat* or abandon* or restor* or rehabilitat* or succession or “soil development”) AND (carbon or “organic matter” or nitrogen)”. Additional searches were conducted with Google Scholar. We examined the abstract of each result to assess its potential to meet the selection criteria for inclusion in the review. The selection criteria were as follows: the articles must compare measurements of soil C (N) concentration, or C (N) density together with bulk density in restored or created and reference (natural) wetlands; the articles must contain quantitative information about soil sampling depth and recovery age at the point when the study began; when more than one data-set was published at the same site but in different studies, only one study was chosen or the data sets were integrated with one another. Under these criteria, we selected 54 study sites from 48 articles (Fig. S2). The articles were read in detail. The reference wetlands were usually adjacent to the restored or created wetlands. The restored or created wetlands were generally of the same hydrogeomorphic type as the reference wetlands. In studies where restored or created wetlands were compared to more than one reference wetland, the average of the references was used. Most studies were conducted in paired plot design and chronosequences using the space-for-time substitution. Very few studies took measurements throughout the progression of the restoration. Because observations in meta-analysis should be independent^55^, only the most recent results were used if more than one measurement was made for the same plot at different times for the same study. For chronosequences, measurements for different recovery ages were considered independent observations if more than one recovery age was reported within a study. In cases where measurements were made at multiple soil depths, only the value for the surface soil layer was used in our analysis.

The data were extracted from the main text, tables, and figures of the articles. The following information was included in the compilation: recovery approaches (restoration and creation), hydrogeomorphic type, recovery age, soil-sampling depth, location (longitude and latitude), climate (mean annual temperature and mean annual precipitation), C pools [i.e., soil organic carbon (SOC) and mineralizable C (MinC)], N pools [i.e., total N (TN) and inorganic N (IN)], phosphorus (P) pools [i.e., total P (TP) and inorganic P (IP)], and other soil parameters [i.e., bulk density (BD), pH, soil texture, soil moisture (SM), cation exchange capacity (CEC), and porosity]. Summary information about the data is available in Table S2. In cases where the studies provided a range of recovery ages, the average age was used. The latitude and longitude of the study site were either reported or obtained from Google Earth according to the location. Data on the mean annual temperature and mean annual precipitation were either reported or obtained from a global climate database (http://www.wordclim.org/) using the latitude and longitude of the study sites. When only data on SOM concentration were reported, the SOM concentration was converted to an SOC concentration by multiplying it by a conversion factor of 0.58^56^. When data on SOC density or total N density together with bulk density were reported, the SOC concentration or total N concentration was calculated using the following formula:

$$SOC(TN)=\frac{SOCD(TND)\times 10}{BD\times H}$$

where SOC and TN are the SOC concentration (g kg^−1^) and total N concentration (g kg^−1^), respectively. The SOCD and TND are the SOC density (Mg ha^−1^) and total N density (Mg ha^−1^), respectively. The BD is the soil bulk density (g cm^−3^) and H is the soil sampling depth (cm).

The constructed database consisted of 409 lines of observations, which were used to explore the general pattern of soil C and N recovery after wetland restoration and creation. To explore the temporal trends in recovery, the recovery ages were categorized into four classes: ≤5, 6–10, 11–20, and >20 years. The soil sampling depths were divided into two groups: ≤10 cm and >10 cm. We also investigated the effects of recovery approach and hydrogeomorphic type on soil C and N recovery. To reduce the influence of recovery age and to include as much data as possible in the analysis, we chose data with recovery ages ≤30 years. The wetlands were classified as “restored” or “created” and grouped into seven hydrogeomorphic classes according to Smith *et al*.^57^, including depressional, riverine, lacustrine, tidal, mineral flat, organic flat, and slope wetlands. Because of the small number of studies on lacustrine, mineral flat and slope wetlands, these types were not analyzed.

### Statistical analyses

In this study, we employed a meta-analysis to determine changes in soil C and N after wetland restoration and creation. Fourteen variables related to soil C and N pools, and other soil physicochemical properties were selected. One common metric of effect size in a meta-analysis is the log response ratio (RR), which is calculated as the ratio of its value in the experimental treatment (Xe) to that in the control treatment (Xc). In our study, Xe is the value of the measured variable in the restored or created wetland, and Xc is the value of the variable in the reference wetland. To improve the statistical performance, the RR was log-transformed such that lnRR = ln (Xe/Xc) = ln (Xe) – ln (Xc). Among the 409 observations in our dataset, only 173 reported standard errors/deviations and sample sizes of the response variables; thus, we used resampling methods for this meta-analysis. Different from the conventional parametric methods, resampling methods do not require computation of the standard errors of the individual effect size estimates^58^. Bootstrapping (9999 iterations) was used to calculate bias-corrected 95% confidence intervals (CIs) around the mean effect size. If the 95% CI of the effect size for a variable did not include zero, the mean value of the variable in the restored and created wetland was considered significantly different from that in the natural wetland. The percentage difference of a variable between the restored and created and natural wetlands was calculated by the following equation: (*e* ^RR++^ − 1) × 100%, where RR_++_ is the mean response ratio. To determine whether there were significant differences in the mean response ratio among various categories, we employed randomization tests to calculate the significance level for between-class heterogeneity (Q_B_). The meta-analysis was conducted using the statistical software Meta-Win^59^. Linear-regression analysis was performed to establish the relationships between lnRR of SOC (total N) and latitude, climate (mean annual temperature and mean annual precipitation), soil texture (clay + silt), and between lnRR of the SOC and total N, lnRR of the soil C:N ratio and recovery age.

## Electronic supplementary material

supplementary information
