# Supplementary material for: A synthesis of soil carbon and nitrogen recovery after wetland restoration and creation in the United States
Source: Sci Rep. 2017 Aug 11;7:7966. doi: 10.1038/s41598-017-08511-y (PMC5554168; doi:10.1038/s41598-017-08511-y)
Supplement: Supplementary file 1 — supplementary information [file 41598_2017_8511_MOESM1_ESM.pdf]

A synthesis of soil carbon and nitrogen recovery after wetland restoration and creation in the United States

Lingfei Yu, Yao Huang, Feifei Sun, Wenjuan Sun*

State Key Laboratory of Vegetation and Environmental Change, Institute of Botany, Chinese Academy of Sciences, Beijing 100093, PR China

*Corresponding author:
E-mail address: sunwj@ibcas.ac.cn (Wenjuan Sun)

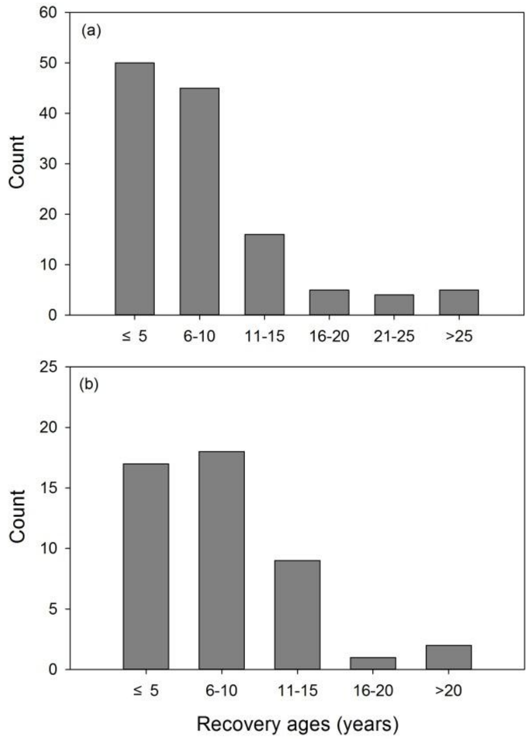


**Figure S1. Frequency distribution of recovery ages for SOC (a) and total N (b).**

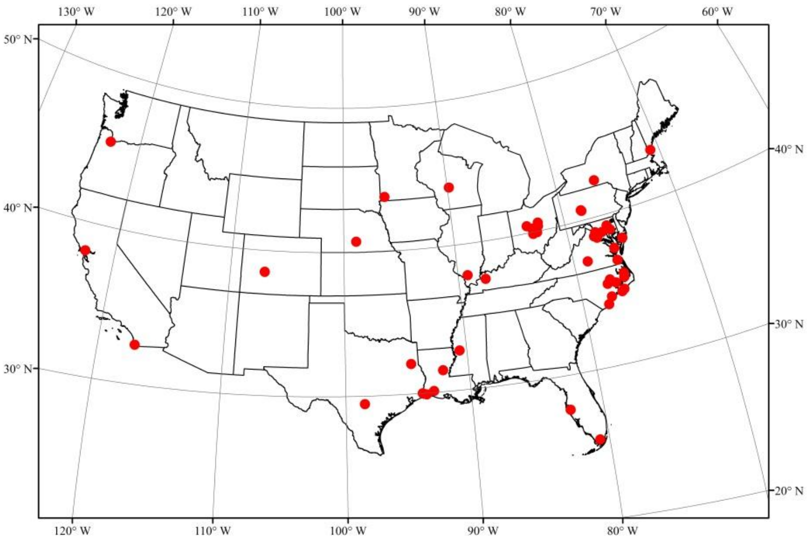


**Figure S2. The distribution of study sites in the conterminous United States.** The map was generated using ESRI ArcMap 10.0 (http://www.esri.com/).

**Table S1. Percentage of difference among 14 variables related to soil carbon, nitrogen, phosphorus and other properties between restored and created wetlands and natural wetlands.** The percentage of difference was calculated as $(e^{RR_{++}} - 1) \times 100\%$, where $RR_{++}$ is the mean response ratio. The data in parentheses represent 95% confidence intervals. SOC, soil organic carbon; MinC, mineralizable carbon; TN, total nitrogen; IN, inorganic nitrogen; TP, total phosphorus; IP, inorganic phosphorus; BD, bulk density; SM, soil moisture; and CEC, cation exchange capacity.

| Variables | Sample size (n) | Percentage of difference |
|---|---|---|
| *Carbon* | | |
| SOC | 125 | -59.43 (-64.8, -53.41) |
| MinC | 6 | -67.02 (-79.61, -45.17) |
| *Nitrogen* | | |
| TN | 47 | -52.33 (-64.05, -40.3) |
| IN | 25 | -51.5 (-69.1, -28.54) |
| *Phosphorus* | | |
| TP | 18 | -5.19 (-44.72, 66.81) |
| IP | 9 | 15.87 (-69.25, 402.1) |
| *Other properties* | | |
| BD | 47 | 80.95 (54.43, 116.6) |
| pH | 41 | 13.98 (9.14, 18.73) |
| Clay | 17 | -4.38 (-28.4, 20.63) |
| Silt | 13 | -11.18 (-44.24, 25.51) |
| Sand | 22 | -7.75 (-31.43, 22.53) |
| SM | 23 | -10.67 (-22.87, 4.86) |
| CEC | 13 | -41.66 (-58.62, -19.19) |
| Porosity | 3 | -18.5 (-30.51, -4.41) |

**Table S2. Summary information for each observation compiled from the literature in this synthesis.** SOC, soil organic carbon; MinC, mineralizable carbon; TN, total nitrogen; IN, inorganic nitrogen; TP, total phosphorus; IP, inorganic phosphorus; BD, bulk density; SM, soil moisture; CEC, cation exchange capacity; MAT, mean annual temperature; and MAP, mean annual precipitation.

| Source Paper | Hydrogeomorphic type | Recovery approach | Variables | Recovery age | Depth (cm) | Location | Latitude | Longitude | MAT (℃) | MAP (mm) |
|---|---|---|---|---|---|---|---|---|---|---|
| Ahn and Peralta, 2012 | depressional | created | pH | 11 | 0-7.5 | Virginia | 38.82 | -77.67 | 12.80 | 993 |
| Ahn and Peralta, 2012 | depressional | created | SOC | 11 | 0-7.5 | Virginia | 38.82 | -77.67 | 12.80 | 993 |
| Ahn and Peralta, 2012 | depressional | created | SN | 11 | 0-7.5 | Virginia | 38.82 | -77.67 | 12.80 | 993 |
| Ahn and Peralta, 2012 | depressional | created | pH | 11 | 0-7.5 | Virginia | 38.82 | -77.67 | 12.80 | 993 |
| Ahn and Peralta, 2012 | depressional | created | SOC | 11 | 0-7.5 | Virginia | 38.82 | -77.67 | 12.80 | 993 |
| Ahn and Peralta, 2012 | depressional | created | SN | 11 | 0-7.5 | Virginia | 38.82 | -77.67 | 12.80 | 993 |
| Ahn and Peralta, 2012 | depressional | created | pH | 11 | 0-7.5 | Virginia | 38.82 | -77.67 | 12.80 | 993 |
| Ahn and Peralta, 2012 | depressional | created | SOC | 11 | 0-7.5 | Virginia | 38.82 | -77.67 | 12.80 | 993 |
| Ahn and Peralta, 2012 | depressional | created | SN | 11 | 0-7.5 | Virginia | 38.82 | -77.67 | 12.80 | 993 |
| Ahn and Peralta, 2012 | depressional | created | pH | 11 | 0-7.5 | Virginia | 38.82 | -77.67 | 12.80 | 993 |
| Ahn and Peralta, 2012 | depressional | created | SOC | 11 | 0-7.5 | Virginia | 38.82 | -77.67 | 12.80 | 993 |
| Ahn and Peralta, 2012 | depressional | created | SN | 11 | 0-7.5 | Virginia | 38.82 | -77.67 | 12.80 | 993 |
| Armitage et al., 2014 | tidal | restored | SOC | 3 | 0-10 | Texas | 30 | -93.85 | 19.88 | 1447 |
| Armitage et al., 2014 | tidal | restored | SOC | 3 | 0-10 | Texas | 30 | -93.85 | 19.88 | 1447 |
| Armitage et al., 2014 | tidal | restored | SOC | 3 | 0-10 | Texas | 30 | -93.85 | 19.88 | 1447 |
| Armitage et al., 2014 | tidal | restored | SN | 3 | 0-10 | Texas | 30 | -93.85 | 19.88 | 1447 |
| Armitage et al., 2014 | tidal | restored | SN | 3 | 0-10 | Texas | 30 | -93.85 | 19.88 | 1447 |
| Armitage et al., 2014 | tidal | restored | SN | 3 | 0-10 | Texas | 30 | -93.85 | 19.88 | 1447 |
| Armitage et al., 2014 | tidal | restored | SP | 3 | 0-10 | Texas | 30 | -93.85 | 19.88 | 1447 |
| Armitage et al., 2014 | tidal | restored | SP | 3 | 0-10 | Texas | 30 | -93.85 | 19.88 | 1447 |
| Armitage et al., 2014 | tidal | restored | SP | 3 | 0-10 | Texas | 30 | -93.85 | 19.88 | 1447 |
| Armitage et al., 2014 | tidal | restored | sand | 3 | 0-10 | Texas | 30 | -93.85 | 19.88 | 1447 |
| Armitage et al., 2014 | tidal | restored | sand | 3 | 0-10 | Texas | 30 | -93.85 | 19.88 | 1447 |
| Armitage et al., 2014 | tidal | restored | sand | 3 | 0-10 | Texas | 30 | -93.85 | 19.88 | 1447 |
| Ballantine and Schneider, 2009 | depressional | restored | SOC | 4 | 0-5 | New York | 42.39 | -76.44 | 7.41 | 941 |
| Ballantine and Schneider, 2009 | depressional | restored | SOC | 12 | 0-5 | New York | 42.39 | -76.44 | 7.41 | 941 |
| Ballantine and Schneider, 2009 | depressional | restored | SOC | 32.5 | 0-5 | New York | 42.39 | -76.44 | 7.41 | 941 |
| Ballantine and Schneider, 2009 | depressional | restored | SOC | 52.5 | 0-5 | New York | 42.39 | -76.44 | 7.41 | 941 |
| Ballantine and Schneider, 2009 | depressional | restored | BD | 4 | 0-5 | New York | 42.39 | -76.44 | 7.41 | 941 |
| Ballantine and Schneider, 2009 | depressional | restored | BD | 12 | 0-5 | New York | 42.39 | -76.44 | 7.41 | 941 |
| Ballantine and Schneider, 2009 | depressional | restored | BD | 32.5 | 0-5 | New York | 42.39 | -76.44 | 7.41 | 941 |
| Ballantine and Schneider, 2009 | depressional | restored | BD | 52.5 | 0-5 | New York | 42.39 | -76.44 | 7.41 | 941 |
| Ballantine and Schneider, 2009 | depressional | restored | CEC | 4 | 0-5 | New York | 42.39 | -76.44 | 7.41 | 941 |

| | | | | | | | | | | |
|---|---|---|---|---|---|---|---|---|---|---|
| Ballantine and Schneider, 2009 | depressional | restored | CEC | 12 | 0-5 | New York | 42.39 | -76.44 | 7.41 | 941 |
| Ballantine and Schneider, 2009 | depressional | restored | CEC | 32.5 | 0-5 | New York | 42.39 | -76.44 | 7.41 | 941 |
| Ballantine and Schneider, 2009 | depressional | restored | CEC | 52.5 | 0-5 | New York | 42.39 | -76.44 | 7.41 | 941 |
| Besasie and Buckley, 2012 | depressional | restored | SOC | 3 | 0-43 | Wisconsin | 44.00 | -89.83 | 6.70 | 812 |
| Besasie and Buckley, 2012 | depressional | restored | SOC | 7.5 | 0-43 | Wisconsin | 44.00 | -89.83 | 6.70 | 812 |
| Besasie and Buckley, 2012 | depressional | restored | SOC | 5.5 | 0-21 | Wisconsin | 44.00 | -89.83 | 6.70 | 812 |
| Bishel-Machung et al., 1996 | | created | SOC | 4.5 | 0-5 | Pennsylvania | 40.64 | -78.27 | 9.01 | 993 |
| Bishel-Machung et al., 1996 | riverine | created | SOC | 4.5 | 0-5 | Pennsylvania | 40.64 | -78.27 | 9.01 | 993 |
| Bishel-Machung et al., 1996 | depressional | created | SOC | 4.5 | 0-5 | Pennsylvania | 40.64 | -78.27 | 9.01 | 993 |
| Broome et al., 1986 | tidal | created | pH | 6 | 0-10 | North Carolina | 34.87 | -76.46 | 16.99 | 1415 |
| Broome et al., 1986 | tidal | created | BD | 6 | 0-10 | North Carolina | 34.87 | -76.46 | 16.99 | 1415 |
| Broome et al., 1986 | tidal | created | SOC | 6 | 0-10 | North Carolina | 34.87 | -76.46 | 16.99 | 1415 |
| Broome et al., 1986 | tidal | created | IN | 6 | 0-10 | North Carolina | 34.87 | -76.46 | 16.99 | 1415 |
| Broome et al., 1986 | tidal | created | SP | 6 | 0-10 | North Carolina | 34.87 | -76.46 | 16.99 | 1415 |
| Bruland and Richardson, 2005 | riverine | restored | SOC | 1 | 0-20 | North Carolina | 35.71 | -77.41 | 15.67 | 1218 |
| Bruland and Richardson, 2005 | riverine | created | SOC | 2 | 0-20 | North Carolina | 35.71 | -77.41 | 15.67 | 1218 |
| Bruland and Richardson, 2005 | mineral soil flat | restored | SOC | 1 | 0-20 | North Carolina | 35.71 | -77.41 | 15.67 | 1218 |
| Bruland and Richardson, 2005 | organic soil flat | restored | SOC | 5 | 0-20 | North Carolina | 35.71 | -77.41 | 15.67 | 1218 |
| Bruland and Richardson, 2005 | riverine | restored | BD | 1 | 0-20 | North Carolina | 35.71 | -77.41 | 15.67 | 1218 |
| Bruland and Richardson, 2005 | riverine | created | BD | 2 | 0-20 | North Carolina | 35.71 | -77.41 | 15.67 | 1218 |
| Bruland and Richardson, 2005 | mineral soil flat | restored | BD | 1 | 0-20 | North Carolina | 35.71 | -77.41 | 15.67 | 1218 |
| Bruland and Richardson, 2005 | organic soil flat | restored | BD | 5 | 0-20 | North Carolina | 35.71 | -77.41 | 15.67 | 1218 |
| Bruland and Richardson, 2005 | riverine | restored | pH | 1 | 0-20 | North Carolina | 35.71 | -77.41 | 15.67 | 1218 |
| Bruland and Richardson, 2005 | riverine | created | pH | 2 | 0-20 | North Carolina | 35.71 | -77.41 | 15.67 | 1218 |
| Bruland and Richardson, 2005 | mineral soil flat | restored | pH | 1 | 0-20 | North Carolina | 35.71 | -77.41 | 15.67 | 1218 |
| Bruland and Richardson, 2005 | organic soil flat | restored | pH | 5 | 0-20 | North Carolina | 35.71 | -77.41 | 15.67 | 1218 |
| Bruland and Richardson, 2005 | riverine | restored | sand | 1 | 0-20 | North Carolina | 35.71 | -77.41 | 15.67 | 1218 |
| Bruland and Richardson, 2005 | riverine | created | sand | 2 | 0-20 | North Carolina | 35.71 | -77.41 | 15.67 | 1218 |
| Bruland and Richardson, 2005 | mineral soil flat | restored | sand | 1 | 0-20 | North Carolina | 35.71 | -77.41 | 15.67 | 1218 |
| Bruland and Richardson, 2006 | riverine | restored | SOC | 2 | 0-20 | North Carolina | 35.46 | -77.67 | 15.84 | 1242 |
| Bruland and Richardson, 2006 | riverine | created | SOC | 7 | 0-20 | North Carolina | 35.46 | -77.67 | 15.84 | 1242 |
| Bruland and Richardson, 2006 | riverine | created | SOC | 3 | 0-20 | North Carolina | 35.46 | -77.67 | 15.84 | 1242 |
| Bruland and Richardson, 2006 | mineral soil flat | restored | SOC | 4 | 0-20 | North Carolina | 35.46 | -77.67 | 15.84 | 1242 |
| Bruland and Richardson, 2006 | mineral soil flat | restored | SOC | 7 | 0-20 | North Carolina | 35.46 | -77.67 | 15.84 | 1242 |
| Bruland and Richardson, 2006 | organic soil flat | restored | SOC | 4 | 0-20 | North Carolina | 35.46 | -77.67 | 15.84 | 1242 |
| Bruland and Richardson, 2006 | organic soil flat | restored | SOC | 7 | 0-20 | North Carolina | 35.46 | -77.67 | 15.84 | 1242 |
| Bruland et al., 2006 | riverine | restored | SM | 1 | 0-20 | North Carolina | 35.71 | -77.41 | 15.67 | 1218 |
| Bruland et al., 2006 | riverine | created | SM | 2 | 0-20 | North Carolina | 35.71 | -77.41 | 15.67 | 1218 |
| Bruland et al., 2006 | mineral soil flat | restored | SM | 1 | 0-20 | North Carolina | 35.71 | -77.41 | 15.67 | 1218 |
| Bruland et al., 2006 | organic soil flat | restored | SM | 5 | 0-20 | North Carolina | 35.71 | -77.41 | 15.67 | 1218 |

| | | | | | | | | | | |
|---|---|---|---|---|---|---|---|---|---|---|
| Bush, 2008 | riverine | restored | SOC | 25 | 0-10 | Texas | 29.45 | -98.49 | 20.62 | 693 |
| Bush, 2008 | riverine | restored | SOC | 39 | 0-10 | Texas | 29.45 | -98.49 | 20.62 | 693 |
| Bush, 2008 | riverine | restored | SOC | 45 | 0-10 | Texas | 29.45 | -98.49 | 20.62 | 693 |
| Bush, 2008 | riverine | restored | SOC | 47 | 0-10 | Texas | 29.45 | -98.49 | 20.62 | 693 |
| Bush, 2008 | riverine | restored | SOC | 49 | 0-10 | Texas | 29.45 | -98.49 | 20.62 | 693 |
| Bush, 2008 | riverine | restored | SOC | 53 | 0-10 | Texas | 29.45 | -98.49 | 20.62 | 693 |
| Bush, 2008 | riverine | restored | SN | 25 | 0-10 | Texas | 29.45 | -98.49 | 20.62 | 693 |
| Bush, 2008 | riverine | restored | SN | 39 | 0-10 | Texas | 29.45 | -98.49 | 20.62 | 693 |
| Bush, 2008 | riverine | restored | SN | 45 | 0-10 | Texas | 29.45 | -98.49 | 20.62 | 693 |
| Bush, 2008 | riverine | restored | SN | 47 | 0-10 | Texas | 29.45 | -98.49 | 20.62 | 693 |
| Bush, 2008 | riverine | restored | SN | 49 | 0-10 | Texas | 29.45 | -98.49 | 20.62 | 693 |
| Bush, 2008 | riverine | restored | SN | 53 | 0-10 | Texas | 29.45 | -98.49 | 20.62 | 693 |
| Callaway et al., 2012 | tidal | restored | SOC | 31.5 | 0-20 | | 38.13 | -122.47 | 14.35 | 736 |
| Callaway et al., 2012 | tidal | restored | BD | 31.5 | 0-20 | | 38.13 | -122.47 | 14.35 | 736 |
| Campbell et al., 2002 | depressional | created | SOC | 5 | 0-20 | Pennsylvania | 40.69 | -78.31 | 7.52 | 1055 |
| Campbell et al., 2002 | depressional | created | BD | 5 | 0-20 | Pennsylvania | 40.69 | -78.31 | 7.52 | 1055 |
| Campbell et al., 2002 | depressional | created | sand | 5 | 0-20 | Pennsylvania | 40.69 | -78.31 | 7.52 | 1055 |
| Campbell et al., 2002 | depressional | created | silt | 5 | 0-20 | Pennsylvania | 40.69 | -78.31 | 7.52 | 1055 |
| Campbell et al., 2002 | depressional | created | clay | 5 | 0-20 | Pennsylvania | 40.69 | -78.31 | 7.52 | 1055 |
| Cole et al., 2001 | depressional | created | SOC | 20 | 0-20 | Pennsylvania | 40.64 | -78.27 | 9.01 | 993 |
| Cole et al., 2001 | depressional | created | SOC | 20 | 0-20 | Pennsylvania | 40.64 | -78.27 | 9.01 | 993 |
| Cole et al., 2001 | riverine | created | SOC | 11 | 0-20 | Pennsylvania | 40.64 | -78.27 | 9.01 | 993 |
| Cole et al., 2001 | slope | created | SOC | 8 | 0-20 | Pennsylvania | 40.64 | -78.27 | 9.01 | 993 |
| Cole et al., 2001 | riverine | created | SOC | 7 | 0-20 | Pennsylvania | 40.64 | -78.27 | 9.01 | 993 |
| Cole et al., 2001 | riverine | created | SOC | 6 | 0-20 | Pennsylvania | 40.64 | -78.27 | 9.01 | 993 |
| Cole et al., 2001 | riverine | created | SOC | 5 | 0-20 | Pennsylvania | 40.64 | -78.27 | 9.01 | 993 |
| Cornell et al., 2007 | tidal | created | SOC | 1 | 0-10 | North Carolina | 34.58 | -77.58 | 16.98 | 1377 |
| Cornell et al., 2007 | tidal | created | SOC | 3 | 0-10 | North Carolina | 34.58 | -77.58 | 16.98 | 1377 |
| Cornell et al., 2007 | tidal | created | SOC | 8 | 0-10 | North Carolina | 34.58 | -77.58 | 16.98 | 1377 |
| Cornell et al., 2007 | tidal | created | SOC | 11 | 0-10 | North Carolina | 34.58 | -77.58 | 16.98 | 1377 |
| Cornell et al., 2007 | tidal | created | SOC | 13 | 0-10 | North Carolina | 34.58 | -77.58 | 16.98 | 1377 |
| Cornell et al., 2007 | tidal | created | SOC | 24 | 0-10 | North Carolina | 34.58 | -77.58 | 16.98 | 1377 |
| Cornell et al., 2007 | tidal | created | SOC | 26 | 0-10 | North Carolina | 34.58 | -77.58 | 16.98 | 1377 |
| Cornell et al., 2007 | tidal | created | SOC | 28 | 0-10 | North Carolina | 34.58 | -77.58 | 16.98 | 1377 |
| Craft et al., 1999 | tidal | created | BD | 24 | 0-10 | North Carolina | 34.12 | -77.95 | 17.28 | 1389 |
| Craft et al., 1999 | tidal | created | SOC | 24 | 0-10 | North Carolina | 34.12 | -77.95 | 17.28 | 1389 |
| Craft et al., 1999 | tidal | created | SN | 24 | 0-10 | North Carolina | 34.12 | -77.95 | 17.28 | 1389 |
| Craft et al., 1999 | tidal | created | SP | 24 | 0-10 | North Carolina | 34.12 | -77.95 | 17.28 | 1389 |
| Craft et al., 1999 | tidal | created | BD | 21 | 0-10 | North Carolina | 34.87 | -76.46 | 16.99 | 1415 |
| Craft et al., 1999 | tidal | created | SOC | 21 | 0-10 | North Carolina | 34.87 | -76.46 | 16.99 | 1415 |

| | | | | | | | | | | |
|---|---|---|---|---|---|---|---|---|---|---|
| Craft et al., 1999 | tidal | created | SN | 21 | 0-10 | North Carolina | 34.87 | -76.46 | 16.99 | 1415 |
| Craft et al., 1999 | tidal | created | SP | 21 | 0-10 | North Carolina | 34.87 | -76.46 | 16.99 | 1415 |
| Craft et al., 2002 | tidal | created | BD | 15 | 0-30 | North Carolina | 35.42 | -76.99 | 16.16 | 1303 |
| Craft et al., 2002 | tidal | created | SOC | 15 | 0-30 | North Carolina | 35.42 | -76.99 | 16.16 | 1303 |
| Craft et al., 2002 | tidal | created | SN | 15 | 0-30 | North Carolina | 35.42 | -76.99 | 16.16 | 1303 |
| Craft et al., 2002 | tidal | created | SP | 15 | 0-30 | North Carolina | 35.42 | -76.99 | 16.16 | 1303 |
| Craft et al., 2002 | tidal | created | pH | 15 | 0-30 | North Carolina | 35.42 | -76.99 | 16.16 | 1303 |
| Craft et al., 2002 | tidal | created | porosity | 15 | 0-30 | North Carolina | 35.42 | -76.99 | 16.16 | 1303 |
| D’Angelo et al., 2005 | riverine | restored | SOC | 7 | 0-6 | Kentucky | 37.41 | -87.66 | 13.95 | 1196 |
| D’Angelo et al., 2005 | riverine | restored | BD | 7 | 0-6 | Kentucky | 37.41 | -87.66 | 13.95 | 1196 |
| Ducey et al., 2015 | organic soil flat | restored | SOC | 5.5 | 0-10 | North Carolina | 35.64 | -76.22 | 16.3 | 1308 |
| Ducey et al., 2015 | organic soil flat | restored | SOC | 5.5 | 0-10 | North Carolina | 35.64 | -76.22 | 16.3 | 1308 |
| Ducey et al., 2015 | organic soil flat | restored | SOC | 5.5 | 0-10 | North Carolina | 35.64 | -76.22 | 16.3 | 1308 |
| Ducey et al., 2015 | organic soil flat | restored | SOC | 5.5 | 0-10 | North Carolina | 35.64 | -76.22 | 16.3 | 1308 |
| Ducey et al., 2015 | organic soil flat | restored | SN | 5.5 | 0-10 | North Carolina | 35.64 | -76.22 | 16.3 | 1308 |
| Ducey et al., 2015 | organic soil flat | restored | SN | 5.5 | 0-10 | North Carolina | 35.64 | -76.22 | 16.3 | 1308 |
| Ducey et al., 2015 | organic soil flat | restored | SN | 5.5 | 0-10 | North Carolina | 35.64 | -76.22 | 16.3 | 1308 |
| Ducey et al., 2015 | organic soil flat | restored | SN | 5.5 | 0-10 | North Carolina | 35.64 | -76.22 | 16.3 | 1308 |
| Ducey et al., 2015 | organic soil flat | restored | pH | 5.5 | 0-10 | North Carolina | 35.64 | -76.22 | 16.3 | 1308 |
| Ducey et al., 2015 | organic soil flat | restored | pH | 5.5 | 0-10 | North Carolina | 35.64 | -76.22 | 16.3 | 1308 |
| Ducey et al., 2015 | organic soil flat | restored | pH | 5.5 | 0-10 | North Carolina | 35.64 | -76.22 | 16.3 | 1308 |
| Ducey et al., 2015 | organic soil flat | restored | pH | 5.5 | 0-10 | North Carolina | 35.64 | -76.22 | 16.3 | 1308 |
| Ducey et al., 2015 | organic soil flat | restored | SM | 5.5 | 0-10 | North Carolina | 35.64 | -76.22 | 16.3 | 1308 |
| Ducey et al., 2015 | organic soil flat | restored | SM | 5.5 | 0-10 | North Carolina | 35.64 | -76.22 | 16.3 | 1308 |
| Ducey et al., 2015 | organic soil flat | restored | SM | 5.5 | 0-10 | North Carolina | 35.64 | -76.22 | 16.3 | 1308 |
| Ducey et al., 2015 | organic soil flat | restored | SM | 5.5 | 0-10 | North Carolina | 35.64 | -76.22 | 16.3 | 1308 |
| Edwards and Proffitt, 2003 | tidal | created | SOC | 19 | 0-10 | Louisiana | 30.10 | -92.93 | 19.80 | 1465 |
| Edwards and Proffitt, 2003 | tidal | created | SOC | 19 | 0-10 | Louisiana | 30.10 | -92.93 | 19.80 | 1465 |
| Edwards and Proffitt, 2003 | tidal | created | SOC | 9 | 0-10 | Louisiana | 30.10 | -92.93 | 19.80 | 1465 |
| Edwards and Proffitt, 2003 | tidal | created | SOC | 9 | 0-10 | Louisiana | 30.10 | -92.93 | 19.80 | 1465 |
| Edwards and Proffitt, 2003 | tidal | created | SOC | 6 | 0-10 | Louisiana | 30.10 | -92.93 | 19.80 | 1465 |
| Edwards and Proffitt, 2003 | tidal | created | SOC | 6 | 0-10 | Louisiana | 30.10 | -92.93 | 19.80 | 1465 |
| Edwards and Proffitt, 2003 | tidal | created | SOC | 3 | 0-10 | Louisiana | 30.10 | -92.93 | 19.80 | 1465 |
| Edwards and Proffitt, 2003 | tidal | created | SOC | 3 | 0-10 | Louisiana | 30.10 | -92.93 | 19.80 | 1465 |
| Edwards and Proffitt, 2003 | tidal | created | BD | 19 | 0-10 | Louisiana | 30.10 | -92.93 | 19.80 | 1465 |
| Edwards and Proffitt, 2003 | tidal | created | BD | 19 | 0-10 | Louisiana | 30.10 | -92.93 | 19.80 | 1465 |
| Edwards and Proffitt, 2003 | tidal | created | BD | 9 | 0-10 | Louisiana | 30.10 | -92.93 | 19.80 | 1465 |
| Edwards and Proffitt, 2003 | tidal | created | BD | 9 | 0-10 | Louisiana | 30.10 | -92.93 | 19.80 | 1465 |
| Edwards and Proffitt, 2003 | tidal | created | BD | 6 | 0-10 | Louisiana | 30.10 | -92.93 | 19.80 | 1465 |
| Edwards and Proffitt, 2003 | tidal | created | BD | 6 | 0-10 | Louisiana | 30.10 | -92.93 | 19.80 | 1465 |

| | | | | | | | | | | |
|---|---|---|---|---|---|---|---|---|---|---|
| Edwards and Proffitt, 2003 | tidal | created | BD | 3 | 0-10 | Louisiana | 30.10 | -92.93 | 19.80 | 1465 |
| Edwards and Proffitt, 2003 | tidal | created | BD | 3 | 0-10 | Louisiana | 30.10 | -92.93 | 19.80 | 1465 |
| Fennessy et al., 2008 | depressional | created | BD | 5 | 0-10 | Ohio | 40.24 | -82.84 | 9.98 | 987 |
| Fennessy et al., 2008 | depressional | created | pH | 5 | 0-10 | Ohio | 40.24 | -82.84 | 9.98 | 987 |
| Fennessy et al., 2008 | depressional | created | IN | 5 | 0-10 | Ohio | 40.24 | -82.84 | 9.98 | 987 |
| Fennessy et al., 2008 | depressional | created | SP | 5 | 0-10 | Ohio | 40.24 | -82.84 | 9.98 | 987 |
| Fennessy et al., 2008 | depressional | created | SOC | 5 | 0-10 | Ohio | 40.24 | -82.84 | 9.98 | 987 |
| Fennessy et al., 2008 | depressional | created | SN | 5 | 0-10 | Ohio | 40.24 | -82.84 | 9.98 | 987 |
| Galatowitsch and van der Valk, 1996 | depressional | restored | pH | 3 | 0-15 | Iowa, Minnesota | 43.77 | -96.05 | 6.83 | 679 |
| Galatowitsch and van der Valk, 1996 | depressional | restored | SOC | 3 | 2-3 | Iowa, Minnesota | 43.77 | -96.05 | 6.83 | 679 |
| Galatowitsch and van der Valk, 1996 | depressional | restored | BD | 3 | 1-2 | Iowa, Minnesota | 43.77 | -96.05 | 6.83 | 679 |
| Giese et al., 2000 | riverine | restored | SOC | 7 | 7.5-15 | South Carolina | | | | |
| Giese et al., 2000 | riverine | restored | SOC | 7 | 7.5-15 | South Carolina | | | | |
| Giese et al., 2000 | riverine | restored | SOC | 11 | 7.5-15 | South Carolina | | | | |
| Gift et al., 2010 | riverine | restored | SOC | 8 | 0-10 | Maryland | 39.25 | -76.50 | 13.37 | 1095 |
| Gift et al., 2010 | riverine | restored | IN | 8 | 0-10 | Maryland | 39.25 | -76.50 | 13.37 | 1095 |
| Gutrich et al., 2009 | depressional | created | SOC | 8 | 0-30 | Colorado | 38.53 | -106.99 | 3.28 | 304 |
| Gutrich et al., 2009 | depressional | created | SOC | 14 | 0-30 | Colorado | 38.52 | -106.99 | 3.28 | 304 |
| Gutrich et al., 2009 | depressional | created | pH | 8 | 0-30 | Colorado | 38.53 | -106.99 | 3.28 | 304 |
| Gutrich et al., 2009 | depressional | created | pH | 14 | 0-30 | Colorado | 38.52 | -106.99 | 3.28 | 304 |
| Gutrich et al., 2009 | depressional | created | SP | 8 | 0-30 | Colorado | 38.53 | -106.99 | 3.28 | 304 |
| Gutrich et al., 2009 | depressional | created | SP | 14 | 0-30 | Colorado | 38.52 | -106.99 | 3.28 | 304 |
| Gutrich et al., 2009 | depressional | created | CEC | 8 | 0-30 | Colorado | 38.53 | -106.99 | 3.28 | 304 |
| Gutrich et al., 2009 | depressional | created | CEC | 14 | 0-30 | Colorado | 38.52 | -106.99 | 3.28 | 304 |
| Hogan et al., 2004 | depressional | restored | SOC | 8 | 0-13 | Maryland | 38.94 | -76.31 | 13.10 | 1590 |
| Hogan et al., 2004 | depressional | restored | BD | 8 | 0-13 | Maryland | 38.94 | -76.31 | 13.10 | 1590 |
| Hogan et al., 2004 | depressional | restored | SN | 8 | 0-13 | Maryland | 38.94 | -76.31 | 13.10 | 1590 |
| Hogan et al., 2004 | depressional | restored | SP | 8 | 0-13 | Maryland | 38.94 | -76.31 | 13.10 | 1590 |
| Hogan et al., 2004 | depressional | restored | IP | 8 | 0-13 | Maryland | 38.94 | -76.31 | 13.10 | 1590 |
| Hogan et al., 2004 | depressional | restored | pH | 8 | 0-13 | Maryland | 38.94 | -76.31 | 13.10 | 1590 |
| Hogan et al., 2004 | depressional | restored | sand | 8 | 0-13 | Maryland | 38.94 | -76.31 | 13.10 | 1590 |
| Hogan et al., 2004 | depressional | restored | clay | 8 | 0-13 | Maryland | 38.94 | -76.31 | 13.10 | 1590 |
| Hogan et al., 2004 | depressional | restored | silt | 8 | 0-13 | Maryland | 38.94 | -76.31 | 13.10 | 1590 |
| Hossler and Bouchard, 2010 | depressional | created | BD | 7 | 0-5 | Ohio | 39.88 | -82.89 | 11.03 | 954 |
| Hossler and Bouchard, 2010 | depressional | created | BD | 3 | 0-5 | Ohio | 40.19 | -82.87 | 10.27 | 978 |
| Hossler and Bouchard, 2010 | depressional | created | BD | 5 | 0-5 | Ohio | 40.35 | -82.33 | 9.47 | 1011 |
| Hossler and Bouchard, 2010 | depressional | created | BD | 8 | 0-5 | Ohio | 40.57 | -82.29 | 8.97 | 1000 |
| Hossler and Bouchard, 2010 | depressional | created | MinC | 7 | 0-5 | Ohio | 39.88 | -82.89 | 11.03 | 954 |

| | | | | | | | | | | |
|---|---|---|---|---|---|---|---|---|---|---|
| Hossler and Bouchard, 2010 | depressional | created | MinC | 3 | 0-5 | Ohio | 40.19 | -82.87 | 10.27 | 978 |
| Hossler and Bouchard, 2010 | depressional | created | MinC | 5 | 0-5 | Ohio | 40.35 | -82.33 | 9.47 | 1011 |
| Hossler and Bouchard, 2010 | depressional | created | MinC | 8 | 0-5 | Ohio | 40.57 | -82.29 | 8.97 | 1000 |
| Hossler and Bouchard, 2010 | depressional | created | SOC | 7 | 0-5 | Ohio | 39.88 | -82.89 | 11.03 | 954 |
| Hossler and Bouchard, 2010 | depressional | created | SOC | 3 | 0-5 | Ohio | 40.19 | -82.87 | 10.27 | 978 |
| Hossler and Bouchard, 2010 | depressional | created | SOC | 5 | 0-5 | Ohio | 40.35 | -82.33 | 9.47 | 1011 |
| Hossler and Bouchard, 2010 | depressional | created | SOC | 8 | 0-5 | Ohio | 40.57 | -82.29 | 8.97 | 1000 |
| Hossler and Bouchard, 2010 | depressional | created | sand | 3 | 0-5 | Ohio | 40.19 | -82.87 | 10.27 | 978 |
| Hossler and Bouchard, 2010 | depressional | created | sand | 5 | 0-5 | Ohio | 40.35 | -82.33 | 9.47 | 1011 |
| Hossler and Bouchard, 2010 | depressional | created | sand | 7 | 0-5 | Ohio | 39.88 | -82.89 | 11.03 | 954 |
| Hossler and Bouchard, 2010 | depressional | created | sand | 8 | 0-5 | Ohio | 40.57 | -82.29 | 8.97 | 1000 |
| Hossler and Bouchard, 2010 | depressional | created | silt | 3 | 0-5 | Ohio | 40.19 | -82.87 | 10.27 | 978 |
| Hossler and Bouchard, 2010 | depressional | created | silt | 5 | 0-5 | Ohio | 40.35 | -82.33 | 9.47 | 1011 |
| Hossler and Bouchard, 2010 | depressional | created | silt | 7 | 0-5 | Ohio | 39.88 | -82.89 | 11.03 | 954 |
| Hossler and Bouchard, 2010 | depressional | created | silt | 8 | 0-5 | Ohio | 40.57 | -82.29 | 8.97 | 1000 |
| Hossler and Bouchard, 2010 | depressional | created | clay | 3 | 0-5 | Ohio | 40.19 | -82.87 | 10.27 | 978 |
| Hossler and Bouchard, 2010 | depressional | created | clay | 5 | 0-5 | Ohio | 40.35 | -82.33 | 9.47 | 1011 |
| Hossler and Bouchard, 2010 | depressional | created | clay | 7 | 0-5 | Ohio | 39.88 | -82.89 | 11.03 | 954 |
| Hossler and Bouchard, 2010 | depressional | created | clay | 8 | 0-5 | Ohio | 40.57 | -82.29 | 8.97 | 1000 |
| Hunter et al., 2008 | riverine | restored | SN | 8 | 0-15 | Louisiana | 31.48 | -92.07 | 18.56 | 1450 |
| Hunter et al., 2008 | riverine | restored | SN | 4.5 | 0-15 | Louisiana | 31.48 | -92.07 | 18.56 | 1450 |
| Hunter et al., 2008 | riverine | restored | MinC | 8 | 0-15 | Louisiana | 31.48 | -92.07 | 18.56 | 1468 |
| Hunter et al., 2008 | riverine | restored | MinC | 4.5 | 0-15 | Louisiana | 31.48 | -92.07 | 18.56 | 1468 |
| Hunter et al., 2008 | riverine | restored | clay | 8 | 0-15 | Louisiana | 31.48 | -92.07 | 18.56 | 1468 |
| Hunter et al., 2008 | riverine | restored | clay | 4.5 | 0-15 | Louisiana | 31.48 | -92.07 | 18.56 | 1468 |
| Hunter et al., 2008 | riverine | restored | silt | 8 | 0-15 | Louisiana | 31.48 | -92.07 | 18.56 | 1468 |
| Hunter et al., 2008 | riverine | restored | silt | 4.5 | 0-15 | Louisiana | 31.48 | -92.07 | 18.56 | 1468 |
| Hunter et al., 2008 | riverine | restored | sand | 8 | 0-15 | Louisiana | 31.48 | -92.07 | 18.56 | 1468 |
| Hunter et al., 2008 | riverine | restored | sand | 4.5 | 0-15 | Louisiana | 31.48 | -92.07 | 18.56 | 1468 |
| Hunter et al., 2008 | riverine | restored | CEC | 8 | 0-15 | Louisiana | 31.48 | -92.07 | 18.56 | 1468 |
| Hunter et al., 2008 | riverine | restored | CEC | 4.5 | 0-15 | Louisiana | 31.48 | -92.07 | 18.56 | 1468 |
| Hunter et al., 2008 | riverine | restored | SM | 8 | 0-15 | Louisiana | 31.48 | -92.07 | 18.56 | 1468 |
| Hunter et al., 2008 | riverine | restored | SM | 4.5 | 0-15 | Louisiana | 31.48 | -92.07 | 18.56 | 1468 |
| Inglett and Inglett, 2013 | organic soil flat | restored | SOC | 2 | 0-10 | Florida | 25.38 | -80.70 | 23.65 | 1381 |
| Inglett and Inglett, 2013 | organic soil flat | restored | SOC | 8 | 0-10 | Florida | 25.38 | -80.70 | 23.65 | 1381 |
| Inglett and Inglett, 2013 | organic soil flat | restored | SOC | 16 | 0-10 | Florida | 25.38 | -80.70 | 23.65 | 1381 |
| Inglett and Inglett, 2013 | organic soil flat | restored | SN | 2 | 0-10 | Florida | 25.38 | -80.70 | 23.65 | 1381 |
| Inglett and Inglett, 2013 | organic soil flat | restored | SN | 8 | 0-10 | Florida | 25.38 | -80.70 | 23.65 | 1381 |
| Inglett and Inglett, 2013 | organic soil flat | restored | SN | 16 | 0-10 | Florida | 25.38 | -80.70 | 23.65 | 1381 |
| Inglett and Inglett, 2013 | organic soil flat | restored | IN | 2 | 0-10 | Florida | 25.38 | -80.70 | 23.65 | 1381 |

| | | | | | | | | | | |
|---|---|---|---|---|---|---|---|---|---|---|
| Inglett and Inglett, 2013 | organic soil flat | restored | IN | 8 | 0-10 | Florida | 25.38 | -80.70 | 23.65 | 1381 |
| Inglett and Inglett, 2013 | organic soil flat | restored | IN | 16 | 0-10 | Florida | 25.38 | -80.70 | 23.65 | 1381 |
| Inglett and Inglett, 2013 | organic soil flat | restored | IN | 2 | 0-10 | Florida | 25.38 | -80.70 | 23.65 | 1381 |
| Inglett and Inglett, 2013 | organic soil flat | restored | IN | 8 | 0-10 | Florida | 25.38 | -80.70 | 23.65 | 1381 |
| Inglett and Inglett, 2013 | organic soil flat | restored | IN | 16 | 0-10 | Florida | 25.38 | -80.70 | 23.65 | 1381 |
| Inglett and Inglett, 2013 | organic soil flat | restored | SP | 2 | 0-10 | Florida | 25.38 | -80.70 | 23.65 | 1381 |
| Inglett and Inglett, 2013 | organic soil flat | restored | SP | 8 | 0-10 | Florida | 25.38 | -80.70 | 23.65 | 1381 |
| Inglett and Inglett, 2013 | organic soil flat | restored | SP | 16 | 0-10 | Florida | 25.38 | -80.70 | 23.65 | 1381 |
| Inglett and Inglett, 2013 | organic soil flat | restored | BD | 2 | 0-10 | Florida | 25.38 | -80.70 | 23.65 | 1381 |
| Inglett and Inglett, 2013 | organic soil flat | restored | BD | 8 | 0-10 | Florida | 25.38 | -80.70 | 23.65 | 1381 |
| Inglett and Inglett, 2013 | organic soil flat | restored | BD | 16 | 0-10 | Florida | 25.38 | -80.70 | 23.65 | 1381 |
| Inglett and Inglett, 2013 | organic soil flat | restored | pH | 2 | 0-10 | Florida | 25.38 | -80.70 | 23.65 | 1381 |
| Inglett and Inglett, 2013 | organic soil flat | restored | pH | 8 | 0-10 | Florida | 25.38 | -80.70 | 23.65 | 1381 |
| Inglett and Inglett, 2013 | organic soil flat | restored | pH | 16 | 0-10 | Florida | 25.38 | -80.70 | 23.65 | 1381 |
| Inglett and Inglett, 2013 | organic soil flat | restored | IP | 2 | 0-10 | Florida | 25.38 | -80.70 | 23.65 | 1381 |
| Inglett and Inglett, 2013 | organic soil flat | restored | IP | 8 | 0-10 | Florida | 25.38 | -80.70 | 23.65 | 1381 |
| Inglett and Inglett, 2013 | organic soil flat | restored | IP | 16 | 0-10 | Florida | 25.38 | -80.70 | 23.65 | 1381 |
| John et al., 2004 | depressional | created | pH | 6 | 0-15 | Texas | 32.08 | -94.62 | 18.02 | 1187 |
| John et al., 2004 | depressional | created | pH | 10 | 0-15 | Texas | 32.08 | -94.62 | 18.02 | 1187 |
| John et al., 2004 | depressional | created | pH | 6 | 0-15 | Texas | 32.08 | -94.62 | 18.02 | 1187 |
| John et al., 2004 | depressional | created | pH | 10 | 0-15 | Texas | 32.08 | -94.62 | 18.02 | 1187 |
| John et al., 2004 | depressional | created | SOC | 6 | 0-15 | Texas | 32.08 | -94.62 | 18.02 | 1187 |
| John et al., 2004 | depressional | created | SOC | 10 | 0-15 | Texas | 32.08 | -94.62 | 18.02 | 1187 |
| John et al., 2004 | depressional | created | SOC | 6 | 0-15 | Texas | 32.08 | -94.62 | 18.02 | 1187 |
| John et al., 2004 | depressional | created | SOC | 10 | 0-15 | Texas | 32.08 | -94.62 | 18.02 | 1187 |
| John et al., 2004 | depressional | created | SN | 6 | 0-15 | Texas | 32.08 | -94.62 | 18.02 | 1187 |
| John et al., 2004 | depressional | created | SN | 10 | 0-15 | Texas | 32.08 | -94.62 | 18.02 | 1187 |
| John et al., 2004 | depressional | created | SN | 6 | 0-15 | Texas | 32.08 | -94.62 | 18.02 | 1187 |
| John et al., 2004 | depressional | created | SN | 10 | 0-15 | Texas | 32.08 | -94.62 | 18.02 | 1187 |
| Kluber et al., 2014 | depressional | restored | SM | 4 | 0-10 | Delaware, Maryland, Virginia and North Carolina | | | | |
| Kluber et al., 2014 | depressional | restored | pH | 4 | 0-10 | Delaware, Maryland, Virginia and North Carolina | | | | |
| Kluber et al., 2014 | depressional | restored | SOC | 4 | 0-10 | Delaware, Maryland, Virginia and North Carolina | | | | |
| Kluber et al., 2014 | depressional | restored | SN | 4 | 0-10 | Delaware, Maryland, Virginia and North Carolina | | | | |
| Langis et al., 1991 | tidal | created | SOC | 4 | 0-8 | California | 32.63 | -117.10 | 15.63 | 834 |
| Langis et al., 1991 | tidal | created | IN | 4 | 0-8 | California | 32.63 | -117.10 | 15.63 | 834 |
| Langis et al., 1991 | tidal | created | IN | 4 | 0-8 | California | 32.63 | -117.10 | 15.63 | 834 |
| Liao et al., 2016 | organic soil flat | restored | BD | 8 | 0-5 | Florida | 25.38 | -80.70 | 23.65 | 1381 |
| Liao et al., 2016 | organic soil flat | restored | BD | 11 | 0-5 | Florida | 25.38 | -80.70 | 23.65 | 1381 |

| | | | | | | | | | | |
|---|---|---|---|---|---|---|---|---|---|---|
| Liao et al., 2016 | organic soil flat | restored | pH | 8 | 0-5 | Florida | 25.38 | -80.70 | 23.65 | 1381 |
| Liao et al., 2016 | organic soil flat | restored | pH | 11 | 0-5 | Florida | 25.38 | -80.70 | 23.65 | 1381 |
| Liao et al., 2016 | organic soil flat | restored | SOC | 8 | 0-5 | Florida | 25.38 | -80.70 | 23.65 | 1381 |
| Liao et al., 2016 | organic soil flat | restored | SOC | 11 | 0-5 | Florida | 25.38 | -80.70 | 23.65 | 1381 |
| Liao et al., 2016 | organic soil flat | restored | SN | 8 | 0-5 | Florida | 25.38 | -80.70 | 23.65 | 1381 |
| Liao et al., 2016 | organic soil flat | restored | SN | 11 | 0-5 | Florida | 25.38 | -80.70 | 23.65 | 1381 |
| Liao et al., 2016 | organic soil flat | restored | SP | 8 | 0-5 | Florida | 25.38 | -80.70 | 23.65 | 1381 |
| Liao et al., 2016 | organic soil flat | restored | SP | 11 | 0-5 | Florida | 25.38 | -80.70 | 23.65 | 1381 |
| Llewellyn and La Peyre, 2011 | tidal | created | SOC | 5 | 0-10 | Louisiana | 29.90 | -93.52 | 19.95 | 1456 |
| Llewellyn and La Peyre, 2011 | tidal | created | SOC | 8 | 0-10 | Louisiana | 29.90 | -93.52 | 19.95 | 1456 |
| Llewellyn and La Peyre, 2011 | tidal | created | SOC | 14 | 0-10 | Louisiana | 29.90 | -93.52 | 19.95 | 1456 |
| Llewellyn and La Peyre, 2011 | tidal | created | SOC | 24 | 0-10 | Louisiana | 29.90 | -93.52 | 19.95 | 1456 |
| Meyer et al., 2008 | depressional | restored | pH | 7 | 0-10 | Nebraska | 40.74 | -98.86 | 9.76 | 645 |
| Meyer et al., 2008 | depressional | restored | CEC | 7 | 0-10 | Nebraska | 40.74 | -98.86 | 9.76 | 645 |
| Meyer et al., 2008 | depressional | restored | pH | 7 | 0-10 | Nebraska | 40.74 | -98.86 | 9.76 | 645 |
| Meyer et al., 2008 | depressional | restored | CEC | 7 | 0-10 | Nebraska | 40.74 | -98.86 | 9.76 | 645 |
| Morgan and Short, 2002 | tidal | restored | SOC | 1 | 0-5 | Maine, New Hampshire | 43.07 | -70.75 | 8.48 | 1131 |
| Morgan and Short, 2002 | tidal | restored | SOC | 2 | 0-5 | Maine, New Hampshire | 43.07 | -70.75 | 8.48 | 1131 |
| Morgan and Short, 2002 | tidal | restored | SOC | 2 | 0-5 | Maine, New Hampshire | 43.07 | -70.75 | 8.48 | 1131 |
| Morgan and Short, 2002 | tidal | restored | SOC | 3 | 0-5 | Maine, New Hampshire | 43.07 | -70.75 | 8.48 | 1131 |
| Morgan and Short, 2002 | tidal | restored | SOC | 6 | 0-5 | Maine, New Hampshire | 43.07 | -70.75 | 8.48 | 1131 |
| Morgan and Short, 2002 | tidal | restored | SOC | 14 | 0-5 | Maine, New Hampshire | 43.07 | -70.75 | 8.48 | 1131 |
| Morse and Bernhardt, 2013 | organic soil flat | restored | BD | 2.5 | 0-20 | North Carolina | 35.91 | -76.16 | 16.6 | 1330 |
| Morse and Bernhardt, 2013 | organic soil flat | restored | pH | 2.5 | 0-20 | North Carolina | 35.91 | -76.16 | 16.6 | 1330 |
| Morse and Bernhardt, 2013 | organic soil flat | restored | SOC | 2.5 | 0-20 | North Carolina | 35.91 | -76.16 | 16.6 | 1330 |
| Morse and Bernhardt, 2013 | organic soil flat | restored | SN | 2.5 | 0-20 | North Carolina | 35.91 | -76.16 | 16.6 | 1330 |
| Moser et al., 2009 | depressional | created | SM | 5.5 | 0-10 | Virginia | 38.82 | -77.67 | 12.49 | 1017 |
| Moser et al., 2009 | depressional | created | SM | 0.5 | 0-10 | Virginia | 38.63 | -77.56 | 13.07 | 999 |
| Moser et al., 2009 | depressional | created | SM | 0.5 | 0-10 | Virginia | 38.63 | -77.56 | 13.07 | 999 |
| Moser et al., 2009 | depressional | created | SN | 5.5 | 0-10 | Virginia | 38.82 | -77.67 | 12.49 | 1017 |
| Moser et al., 2009 | depressional | created | SN | 0.5 | 0-10 | Virginia | 38.63 | -77.56 | 13.07 | 999 |
| Moser et al., 2009 | depressional | created | SN | 0.5 | 0-10 | Virginia | 38.63 | -77.56 | 13.07 | 999 |
| Moser et al., 2009 | depressional | created | IP | 5.5 | 0-10 | Virginia | 38.82 | -77.67 | 12.49 | 1017 |
| Moser et al., 2009 | depressional | created | IP | 0.5 | 0-10 | Virginia | 38.63 | -77.56 | 13.07 | 999 |
| Moser et al., 2009 | depressional | created | IP | 0.5 | 0-10 | Virginia | 38.63 | -77.56 | 13.07 | 999 |
| Moser et al., 2009 | depressional | created | IN | 5.5 | 0-10 | Virginia | 38.82 | -77.67 | 12.49 | 1017 |
| Moser et al., 2009 | depressional | created | IN | 0.5 | 0-10 | Virginia | 38.63 | -77.56 | 13.07 | 999 |

| | | | | | | | | | | |
|---|---|---|---|---|---|---|---|---|---|---|
| Moser et al., 2009 | depressional | created | IN | 0.5 | 0-10 | Virginia | 38.63 | -77.56 | 13.07 | 999 |
| Moser et al., 2009 | depressional | created | IN | 5.5 | 0-10 | Virginia | 38.82 | -77.67 | 12.49 | 1017 |
| Moser et al., 2009 | depressional | created | IN | 0.5 | 0-10 | Virginia | 38.63 | -77.56 | 13.07 | 999 |
| Moser et al., 2009 | depressional | created | IN | 0.5 | 0-10 | Virginia | 38.63 | -77.56 | 13.07 | 999 |
| Moy and Levin, 1991 | tidal | created | SOC | 3 | 0-4 | North Carolina | 34.75 | -76.67 | 17.06 | 1389 |
| Moy and Levin, 1991 | tidal | created | SOC | 3 | 0-4 | North Carolina | 34.75 | -76.67 | 17.06 | 1389 |
| Moy and Levin, 1991 | tidal | created | SOC | 3 | 0-4 | North Carolina | 34.75 | -76.67 | 17.06 | 1389 |
| Orr et al., 2007 | riverine | restored | SM | 1.5 | 0-10 | Wisconsin | | | | |
| Orr et al., 2007 | riverine | restored | SM | 1.5 | 0-10 | Wisconsin | | | | |
| Orr et al., 2007 | riverine | restored | SM | 1.5 | 0-10 | Wisconsin | | | | |
| Orr et al., 2007 | riverine | restored | SM | 1.5 | 0-10 | Wisconsin | | | | |
| Orr et al., 2007 | riverine | restored | SOC | 1.5 | 0-10 | Wisconsin | | | | |
| Orr et al., 2007 | riverine | restored | SOC | 1.5 | 0-10 | Wisconsin | | | | |
| Orr et al., 2007 | riverine | restored | SOC | 1.5 | 0-10 | Wisconsin | | | | |
| Orr et al., 2007 | riverine | restored | SOC | 1.5 | 0-10 | Wisconsin | | | | |
| Osland et al., 2012 | tidal | created | SOC | 8.5 | 0-10 | Florida | 27.68 | -82.53 | 22.45 | 1247 |
| Osland et al., 2012 | tidal | created | SN | 8.5 | 0-10 | Florida | 27.68 | -82.53 | 22.45 | 1247 |
| Osland et al., 2012 | tidal | created | BD | 8.5 | 0-10 | Florida | 27.68 | -82.53 | 22.45 | 1247 |
| Osland et al., 2012 | tidal | created | sand | 8.5 | 0-10 | Florida | 27.68 | -82.53 | 22.45 | 1247 |
| Osland et al., 2012 | tidal | created | SM | 8.5 | 0-10 | Florida | 27.68 | -82.53 | 22.45 | 1247 |
| Osland et al., 2012 | tidal | created | pH | 8.5 | 0-10 | Florida | 27.68 | -82.53 | 22.45 | 1247 |
| Peralta et al., 2013 | depressional | created | pH | 7 | 0-7.5 | Virginia | 38.85 | -77.53 | 12.80 | 993 |
| Peralta et al., 2013 | depressional | created | pH | 3 | 0-7.5 | Virginia | 39.02 | -77.60 | 12.51 | 999 |
| Peralta et al., 2013 | depressional | created | SOC | 7 | 0-7.5 | Virginia | 38.85 | -77.53 | 12.80 | 993 |
| Peralta et al., 2013 | depressional | created | SN | 7 | 0-7.5 | Virginia | 38.85 | -77.53 | 12.80 | 993 |
| Peralta et al., 2013 | depressional | created | SOC | 3 | 0-7.5 | Virginia | 39.02 | -77.60 | 12.51 | 999 |
| Peralta et al., 2013 | depressional | created | SN | 3 | 0-7.5 | Virginia | 39.02 | -77.60 | 12.51 | 999 |
| Shaffer and Ernst, 1999 | slope | | SOC | 6 | 0-5 | Oregon | 45.46 | -122.78 | 11.4 | 1015 |
| Shaffer and Ernst, 1999 | riverine | | SOC | 6 | 0-5 | Oregon | 45.46 | -122.78 | 11.4 | 1015 |
| Shaffer and Ernst, 1999 | depressional | | SOC | 6 | 0-5 | Oregon | 45.46 | -122.78 | 11.4 | 1015 |
| Shaffer and Ernst, 1999 | depressional | | SOC | 6 | 0-5 | Oregon | 45.46 | -122.78 | 11.4 | 1015 |
| Shaffer and Ernst, 1999 | depressional | | SOC | 6 | 0-5 | Oregon | 45.46 | -122.78 | 11.4 | 1015 |
| Spieles et al., 2006 | depressional | created | BD | 11 | 0-10 | Ohio | 39.94 | -82.52 | 10.4 | 1015 |
| Spieles et al., 2006 | depressional | created | SOC | 11 | 0-10 | Ohio | 39.94 | -82.52 | 10.4 | 1015 |
| Spieles et al., 2006 | depressional | restored | BD | 10 | 0-10 | Ohio | 40.48 | -83.32 | 10 | 921 |
| Spieles et al., 2006 | depressional | restored | SOC | 10 | 0-10 | Ohio | 40.48 | -83.32 | 10 | 921 |
| Stolt et al., 2000 | riverine | created | SOC | 7 | 5-15 | Virginia | 37.25 | -78.83 | 13.14 | 1100 |
| Stolt et al., 2000 | riverine | created | SOC | 4 | 5-15 | Virginia | 36.85 | -76.39 | 15.22 | 1144 |
| Stolt et al., 2000 | riverine | created | SOC | 4 | 5-15 | Virginia | 37.67 | -76.38 | 14.37 | 1074 |
| Stolt et al., 2000 | riverine | created | SN | 7 | 5-15 | Virginia | 37.25 | -78.83 | 12.80 | 1098 |

| | | | | | | | | | | |
|---|---|---|---|---|---|---|---|---|---|---|
| Stolt et al., 2000 | riverine | created | SN | 4 | 5-15 | Virginia | 36.85 | -76.39 | 15.00 | 1146 |
| Stolt et al., 2000 | riverine | created | SN | 4 | 5-15 | Virginia | 37.67 | -76.38 | 14.10 | 1093 |
| Stolt et al., 2000 | riverine | created | CEC | 7 | 5-15 | Virginia | 37.25 | -78.83 | 13.14 | 1100 |
| Stolt et al., 2000 | riverine | created | CEC | 4 | 5-15 | Virginia | 36.85 | -76.39 | 15.22 | 1144 |
| Stolt et al., 2000 | riverine | created | CEC | 4 | 5-15 | Virginia | 37.67 | -76.38 | 14.37 | 1074 |
| Stolt et al., 2000 | riverine | created | pH | 7 | 5-15 | Virginia | 37.25 | -78.83 | 13.14 | 1100 |
| Stolt et al., 2000 | riverine | created | pH | 4 | 5-15 | Virginia | 36.85 | -76.39 | 15.22 | 1144 |
| Stolt et al., 2000 | riverine | created | pH | 4 | 5-15 | Virginia | 37.67 | -76.38 | 14.37 | 1074 |
| Stolt et al., 2000 | riverine | created | sand | 7 | 5-15 | Virginia | 37.25 | -78.83 | 13.14 | 1100 |
| Stolt et al., 2000 | riverine | created | sand | 4 | 5-15 | Virginia | 36.85 | -76.39 | 15.22 | 1144 |
| Stolt et al., 2000 | riverine | created | sand | 4 | 5-15 | Virginia | 37.67 | -76.38 | 14.37 | 1074 |
| Stolt et al., 2000 | riverine | created | silt | 7 | 5-15 | Virginia | 37.25 | -78.83 | 13.14 | 1100 |
| Stolt et al., 2000 | riverine | created | silt | 4 | 5-15 | Virginia | 36.85 | -76.39 | 15.22 | 1144 |
| Stolt et al., 2000 | riverine | created | silt | 4 | 5-15 | Virginia | 37.67 | -76.38 | 14.37 | 1074 |
| Stolt et al., 2000 | riverine | created | clay | 7 | 5-15 | Virginia | 37.25 | -78.83 | 13.14 | 1100 |
| Stolt et al., 2000 | riverine | created | clay | 4 | 5-15 | Virginia | 36.85 | -76.39 | 15.22 | 1144 |
| Stolt et al., 2000 | riverine | created | clay | 4 | 5-15 | Virginia | 37.67 | -76.38 | 14.37 | 1074 |
| Taylor and Middleton, 2004 | depressional | created | SOC | 5 | 5-15 | Illinois | 37.83 | -89.17 | 12.9 | 1116 |
| Taylor and Middleton, 2004 | depressional | created | pH | 5 | 5-15 | Illinois | 37.83 | -89.17 | 12.9 | 1116 |
| Ullah and Faulkner, 2006 | riverine | restored | BD | 13 | 0-10 | Mississippi | 32.73 | -90.58 | 17.77 | 1378 |
| Ullah and Faulkner, 2006 | riverine | restored | BD | 13 | 0-10 | Mississippi | 32.73 | -90.58 | 17.77 | 1378 |
| Ullah and Faulkner, 2006 | riverine | restored | SN | 13 | 0-10 | Mississippi | 32.73 | -90.58 | 17.77 | 1378 |
| Ullah and Faulkner, 2006 | riverine | restored | SN | 13 | 0-10 | Mississippi | 32.73 | -90.58 | 17.77 | 1378 |
| Ullah and Faulkner, 2006 | riverine | restored | pH | 13 | 0-10 | Mississippi | 32.73 | -90.58 | 17.77 | 1378 |
| Ullah and Faulkner, 2006 | riverine | restored | pH | 13 | 0-10 | Mississippi | 32.73 | -90.58 | 17.77 | 1378 |
| Ullah and Faulkner, 2006 | riverine | restored | IN | 13 | 0-10 | Mississippi | 32.73 | -90.58 | 17.77 | 1378 |
| Ullah and Faulkner, 2006 | riverine | restored | IN | 13 | 0-10 | Mississippi | 32.73 | -90.58 | 17.77 | 1378 |
| Ullah and Faulkner, 2006 | riverine | restored | IN | 13 | 0-10 | Mississippi | 32.73 | -90.58 | 17.77 | 1378 |
| Ullah and Faulkner, 2006 | riverine | restored | IN | 13 | 0-10 | Mississippi | 32.73 | -90.58 | 17.77 | 1378 |
| Ullah and Faulkner, 2006 | riverine | restored | clay | 13 | 0-10 | Mississippi | 32.73 | -90.58 | 17.77 | 1378 |
| Ullah and Faulkner, 2006 | riverine | restored | clay | 13 | 0-10 | Mississippi | 32.73 | -90.58 | 17.77 | 1378 |
| Ullah and Faulkner, 2006 | riverine | restored | silt | 13 | 0-10 | Mississippi | 32.73 | -90.58 | 17.77 | 1378 |
| Ullah and Faulkner, 2006 | riverine | restored | silt | 13 | 0-10 | Mississippi | 32.73 | -90.58 | 17.77 | 1378 |
| Ullah and Faulkner, 2006 | riverine | restored | porosity | 13 | 0-10 | Mississippi | 32.73 | -90.58 | 17.77 | 1378 |
| Ullah and Faulkner, 2006 | riverine | restored | porosity | 13 | 0-10 | Mississippi | 32.73 | -90.58 | 17.77 | 1378 |
| Verhoeven et al., 2001 | riverine | restored | SP | 2 | 0-30 | Maryland | 38.91 | -77.04 | 13.12 | 1030 |
| Verhoeven et al., 2001 | riverine | restored | SP | 2 | 0-30 | Maryland | 38.18 | -75.49 | 13.40 | 1085 |
| Verhoeven et al., 2001 | riverine | restored | IN | 2 | 0-30 | Maryland | 38.91 | -77.04 | 13.12 | 1030 |
| Verhoeven et al., 2001 | riverine | restored | IN | 2 | 0-30 | Maryland | 38.18 | -75.49 | 13.40 | 1085 |
| Verhoeven et al., 2001 | riverine | restored | IN | 2 | 0-30 | Maryland | 38.91 | -77.04 | 13.12 | 1030 |

| | | | | | | | | | | |
|---|---|---|---|---|---|---|---|---|---|---|
| Verhoeven et al., 2001 | riverine | restored | IN | 2 | 0-30 | Maryland | 38.18 | -75.49 | 13.40 | 1085 |
| Verhoeven et al., 2001 | riverine | restored | IP | 2 | 0-30 | Maryland | 38.91 | -77.04 | 13.12 | 1030 |
| Verhoeven et al., 2001 | riverine | restored | IP | 2 | 0-30 | Maryland | 38.18 | -75.49 | 13.40 | 1085 |
| Verhoeven et al., 2001 | riverine | restored | SOC | 2 | 0-30 | Maryland | 38.91 | -77.04 | 13.12 | 1030 |
| Verhoeven et al., 2001 | riverine | restored | SN | 2 | 0-30 | Maryland | 38.91 | -77.04 | 13.12 | 1030 |
| Verhoeven et al., 2001 | riverine | restored | BD | 2 | 0-30 | Maryland | 38.91 | -77.04 | 13.12 | 1030 |
| Verhoeven et al., 2001 | riverine | restored | SOC | 2 | 0-30 | Maryland | 38.18 | -75.49 | 13.40 | 1085 |
| Verhoeven et al., 2001 | riverine | restored | SN | 2 | 0-30 | Maryland | 38.18 | -75.49 | 13.40 | 1085 |
| Verhoeven et al., 2001 | riverine | restored | BD | 2 | 0-30 | Maryland | 38.18 | -75.49 | 13.40 | 1085 |
| Wolf et al., 2011 | depressional | created | BD | 4 | 0-5 | Virginia | 38.77 | -77.78 | 12.18 | 1041 |
| Wolf et al., 2011 | depressional | created | SN | 4 | 0-5 | Virginia | 38.77 | -77.78 | 12.18 | 1041 |
| Wolf et al., 2011 | depressional | created | SOC | 4 | 0-5 | Virginia | 38.77 | -77.78 | 12.18 | 1041 |
| Wolf et al., 2011 | depressional | created | BD | 10 | 0-5 | Virginia | 38.82 | -77.67 | 12.49 | 1017 |
| Wolf et al., 2011 | depressional | created | SN | 10 | 0-5 | Virginia | 38.82 | -77.67 | 12.49 | 1017 |
| Wolf et al., 2011 | depressional | created | SOC | 10 | 0-5 | Virginia | 38.82 | -77.67 | 12.49 | 1017 |
| Wolf et al., 2011 | depressional | created | BD | 3 | 0-5 | Virginia | 39.02 | -77.60 | 12.51 | 999 |
| Wolf et al., 2011 | depressional | created | SN | 3 | 0-5 | Virginia | 39.02 | -77.60 | 12.51 | 999 |
| Wolf et al., 2011 | depressional | created | SOC | 3 | 0-5 | Virginia | 39.02 | -77.60 | 12.51 | 999 |
| Wolf et al., 2011 | depressional | created | BD | 7 | 0-5 | Virginia | 38.85 | -77.53 | 12.80 | 993 |
| Wolf et al., 2011 | depressional | created | SN | 7 | 0-5 | Virginia | 38.85 | -77.53 | 12.80 | 993 |
| Wolf et al., 2011 | depressional | created | SOC | 7 | 0-5 | Virginia | 38.85 | -77.53 | 12.80 | 993 |
| Wolf et al., 2011 | depressional | created | SM | 3 | 0-5 | Virginia | 39.02 | -77.60 | 12.51 | 999 |
| Wolf et al., 2011 | depressional | created | SM | 4 | 0-5 | Virginia | 38.85 | -77.53 | 12.80 | 993 |
| Wolf et al., 2011 | depressional | created | SM | 7 | 0-5 | Virginia | 38.85 | -77.53 | 12.80 | 993 |
| Wolf et al., 2011 | depressional | created | SM | 10 | 0-5 | Virginia | 38.85 | -77.53 | 12.80 | 993 |
| Wolf et al., 2011 | depressional | created | sand | 3 | 0-5 | Virginia | 39.02 | -77.60 | 12.51 | 999 |
| Wolf et al., 2011 | depressional | created | sand | 4 | 0-5 | Virginia | 38.85 | -77.53 | 12.80 | 993 |
| Wolf et al., 2011 | depressional | created | sand | 7 | 0-5 | Virginia | 38.85 | -77.53 | 12.80 | 993 |
| Wolf et al., 2011 | depressional | created | sand | 10 | 0-5 | Virginia | 38.85 | -77.53 | 12.80 | 993 |
| Wolf et al., 2011 | depressional | created | clay | 3 | 0-5 | Virginia | 39.02 | -77.60 | 12.51 | 999 |
| Wolf et al., 2011 | depressional | created | clay | 4 | 0-5 | Virginia | 38.85 | -77.53 | 12.80 | 993 |
| Wolf et al., 2011 | depressional | created | clay | 7 | 0-5 | Virginia | 38.85 | -77.53 | 12.80 | 993 |
| Wolf et al., 2011 | depressional | created | clay | 10 | 0-5 | Virginia | 38.85 | -77.53 | 12.80 | 993 |
| Zedler and Callaway, 1999 | tidal | created | SOC | 11 | 0-8 | California | 32.63 | -117.10 | 15.63 | 834 |
| Zedler and Callaway, 1999 | tidal | created | SN | 11 | 0-8 | California | 32.63 | -117.10 | 15.63 | 834 |
